# Microsatellite instability test using peptide nucleic acid probe-mediated melting point analysis: a comparison study

**DOI:** 10.1186/s12885-018-5127-6

**Published:** 2018-12-04

**Authors:** Mi Jang, Yujin Kwon, Hoguen Kim, Hyunki Kim, Byung Soh Min, Yehyun Park, Tae Il Kim, Sung Pil Hong, Won Kyu Kim

**Affiliations:** 1 Department of Pathology, Yonsei University College of Medicine, 50 Yonsei-ro, Seodaemun-gu, Seoul, 120-752 South Korea; 2 Brain Korea 21 PLUS Projects for Medical Science, Yonsei University College of Medicine, 50 Yonsei-ro, Seodaemun-gu, Seoul, 120-752 South Korea; 3 Department of Surgery, Yonsei University College of Medicine, Seoul, 120-752 South Korea; 4 Department of Internal Medicine, Institute of Gastroenterology, Yonsei University College of Medicine, Seoul, 120-752 South Korea; 5 Department of Surgery and Cancer, Imperial College London, London, W120NN UK

**Keywords:** Microsatellite instability, Colorectal cancer, Real-time polymerase chain reaction, Peptide nucleic acid probe and immunohistochemistry

## Abstract

**Background:**

Analysis of high microsatellite instability (MSI-H) phenotype in colorectal carcinoma (CRC) is important for evaluating prognosis and choosing a proper adjuvant therapy. Although the conventional MSI analysis methods such as polymerase chain reaction (PCR) fragment analysis and immunohistochemistry (IHC) show high specificity and sensitivity, there are substantial barriers to their use.

**Methods:**

In this study, we analyzed the MSI detection performance of three molecular tests and IHC. For the molecular tests, we included a recently developed peptide nucleic acid probe (PNA)-mediated real-time PCR-based method using five quasi-monomorphic mononucleotide repeat markers (PNA method) and two conventional PCR fragment analysis methods using NCI markers (NCI method) or five quasi-monomorphic mononucleotide repeat markers (MNR method). IHC analysis was performed with four mismatch repair proteins. The performance of each method was validated in 166 CRC patient samples, which consisted of 76 MSI-H and 90 microsatellite stable (MSS) CRCs previously diagnosed by NCI method.

**Results:**

Of the 166 CRCs, 76 MSI-H and 90 MSS CRCs were determined by PNA method. On the other hand, 75 MSI-H and 91 MSS CRCs were commonly determined by IHC and MNR methods. Based on the originally diagnosed MSI status, PNA showed 100% sensitivity and 100% specificity while IHC and MNR showed 98.68% sensitivity and 100% specificity. When we analyzed the maximum sensitivity of MNR and PNA method, which used the same five markers, PNA method could detect alterations in all five mononucleotide repeat markers in samples containing down to 5% MSI-H DNAs, whereas MNR required at least 20% MSI-H DNAs to achieve the same performance.

**Conclusions:**

Based on these findings, we suggest that PNA method can be used as a practical laboratory test for the diagnosis of MSI.

**Electronic supplementary material:**

The online version of this article (10.1186/s12885-018-5127-6) contains supplementary material, which is available to authorized users.

## Background

The molecular pathogenesis of colorectal carcinoma (CRC) is well understood in comparison with other cancers. The development of a broad range of CRCs can be explained by the multistep carcinogenesis model and high microsatellite instability (MSI-H) resulting from deficiencies of the mismatch repair (MMR) gene set, which consists of *MSH2*, *MLH1*, *MSH6*, and *PMS2*. The MSI-H phenotype is found in both hereditary non-polyposis colorectal cancer (HNPCC) with germline mutation in MMR gene set (3%) and sporadic CRCs with CpG island methylator phenotype in MMR gene set (12%), together which account for approximately 15% of all CRCs [1]. Regardless of the origin (hereditary or sporadic) or type of mutation, MSI-H quantitatively or qualitatively alters the expression of numerous genes harboring nucleotide repeats, such as transforming growth factor-β receptor 2, *TCF-4, BAX, MLH3,* and *RAD50*, which may contribute to the development of CRCs, increased neoantigen production, and increased sensitivity to immunotherapy [2–5].

In addition to CRCs, MSI-H has frequently been reported in other types of cancers including endometrial and gastric cancer and is expected to play direct or indirect roles in the development of these cancers. A recent comprehensive MSI screening study showed that the degree of MSI positively correlates with the survival of patients with various cancers [6]. Thus, precise and rapid detection of MSI status has become more crucial for both research and clinical practice. There are several laboratory tests for determining MSI status, including polymerase chain reaction (PCR)-based analysis of MSI markers and immunohistochemistry (IHC) staining of MMR proteins [7]. However, conventional PCR-based MSI interrogation requires complicated steps and additional equipment and shows low sensitivity for samples with a small proportion of tumor cells. On the other hand, IHC analysis requires pathologists, and interpretation criteria can be subjective and affected by technical factors. Therefore, a more accurate and simple MSI test strategy is needed.

We aimed to evaluate the MSI detection performance of various MSI detection methods, including the following: a recently developed peptide nucleotide acid probe (PNA)-mediated real-time PCR-based MSI test using five quasi-monomorphic mononucleotide repeat markers (PNA method) and two conventional PCR fragment analysis methods, PCR fragment analysis with two mononucleotide and three dinucleotide repeat markers proposed by the National Cancer Institute (NCI method) [8], and PCR fragment analysis with the same mononucleotide repeat (MNR) markers as PNA method (MNR method) as well as IHC method. Each MSI detection method was validated in 166 CRCs and paired normal specimens. In this study, we provided practical data for proper selection and application of MSI detection methods.

## Methods

### Cell lines and patient tissue samples

For testing the sensitivity of PNA (U-TOP™ MSI Detection Kit) and MNR (Promega MSI Analysis System Kit), HeLa (microsatellite stable, MSS) and SNU-638 (MSI-H) cell lines were used. HeLa cells and SNU-638 cells were cultured with DMEM and RPMI1640 medium, respectively. Each medium was supplemented with 10% FBS (Invitrogen Life Technologies, Carlsbad, CA, USA) and 1% penicillin/streptomycin.

Formalin-fixed paraffin-embedded (FFPE) tissue samples were collected from 2263 patients with CRCs who visited Severance Hospital between January 2005 and December 2015. A total of 166 CRCs (76 diagnosed as MSI-H and 90 diagnosed as MSS) and matched non-neoplastic colon mucosal tissues were randomly selected for this study. MSI status was previously analyzed using the NCI method. The specimens were obtained from the archives of the Department of Pathology, Yonsei University, Seoul, Korea and the Liver Cancer Specimen Bank of the National Research Resource Bank Program of the Korean Science and Engineering Foundation, Ministry of Science and Technology.

### PNA probe-mediated real-time PCR sensing for detection of MSI status

We tested the performance of the PNA method in detecting MSI status in colon cancer samples using genomic DNA samples (gDNAs) extracted from FFPE CRCs and matched normal tissues. gDNA was isolated according to the manufacturer’s instructions (QIAamp DNA FFPE Tissue Kit, Qiagen, Venlo, Netherlands; Maxwell® 16 FFPE Purification Kit for DNA, Promega). Both quality and quantity of extracted genomic DNA samples were evaluated by using the Nanodrop (Thermo Waltham, MA, USA) and subsequent gel electrophoresis. In PNA method, wild type PNA probe perfectly hybridized with wild type allele, while partial or mismatch hybridization caused melting temperature of PNA probe to be lower than that of perfectly matched probe. At denaturation temperature, PNA probe was subject to fluorescence quenching by random coiling, and this quenching temperature was analyzed by real-time PCR machine [9, 10].

To assess MSI status with PNA method, U-TOP™ MSI Detection Kit was purchased from SeaSun Biomaterials (Daejeon, Korea). This commercially available product employed five MSI marker genes (*NR21*, *NR24*, *NR27, BAT25*, and *BAT26*). A 20-μl mixture composed of gDNA sample (3 μl), 2 × qPCR Premix (10 μl), and dual-labeled (fluorescence and quencher) PNA probes for *NR21*, *NR24*, and *BAT26* (MSI Marker A; 7 μl), as well as another 20-μl mixture composed of gDNA sample (3 μl), 2 × qPCR Premix (10 μl) and dual-labeled PNA probes for *BAT25* and *NR27* (MSI Marker B; 7 μl). The qPCR premix contained dNTP and DNA Taq polymerase, as well as Uracil DNA Glycosylase (UDG). Each PNA probe was fluorescently labeled with Texas-Red, Hexachloro-fluorescein, or Fluorescein amidite. Two individual real-time PCR reactions were performed for each normal and cancer sample using a CFX96 PCR machine (Bio-Rad, Hercules, CA, USA). PCR reaction steps consisted of amplification and subsequent melting point analysis. The amplification condition was 50 °C for 5 min, 95 °C for 10 min, and 60 cycles of 95 °C for 30 s, 65 °C for 30 s, 55 °C for 30 s, and 57 °C for 45 s. The initial incubation at 50 °C for 5 min was required to activate Uracil DNA Glycosylase and prevent possible carryover contamination. In 60 cycles of PCR, four different temperatures and respective optimal times were required. The main purpose of using these conditions was to ensure specific binding of PNA probes to their target MSI marker. In detail, 95 °C for 30 s was for denaturation of template DNAs, 65 °C for 30 s was for binding of PNA probes to their target MSI markers, 55 °C for 30 s was for annealing temperature of primers, and 57 °C for 45 s was for elongation of a polymerase. The melting point analysis condition was 10 min at 95 °C for denaturation and touchdown PCR (90 °C to 45 °C, decreasing 1 °C per cycle). Fluorescence was measured for 10 s at each cycle of touchdown PCR. Obtained melting peaks were analyzed to detect alterations in the five MSI marker genes. A CRC sample was considered to be unstable in a MSI marker gene when a > 3 °C melting temperature difference between a CRC and the normal sample was observed. For the analysis of minimal base pair alteration that PNA method can detect, we used artificially synthesized oligo targets using either deletions or insertions (Macrogen, Seoul, Korea).

### PCR fragment analysis

For NCI method, five microsatellite loci (*BAT-25, BAT-26, D2S123, D5S346,* and *D17S250*) recommended by the 1997 NCI-sponsored MSI workshop were amplified in a single multiplex PCR reaction. PCR products were analyzed by capillary electrophoresis. For interpretation, instability at more than one locus was defined as MSI-H, instability at a single locus was defined as low MSI (MSI-L), and no instability at any locus was defined as MSS. For MNR method, amplification of five MSI markers (*NR21*, *NR24*, *BAT26*, *BAT25,* and *NR27*) was performed using gDNAs extracted from CRCs and matched normal samples. PCR reactions were performed with the MSI Analysis System Kit referring to the MSI Analysis System Version 1.2 protocol. This kit was purchased from Promega (Madison, WI, USA). Amplified PCR products were purified using a PCR clean-up kit (Macherey-Nagel, Düren, Germany), and the size of PCR amplicons was analyzed using an ABI PRISM 3100 Genetic Analyzer (Applied Biosystems, Foster City, CA, USA). Based on data obtained from the sequencer, MSI status was determined by random experts (Macrogen).

### Immunohistochemistry

Paraffin-embedded tissue blocks were cut into 4-μm sections. IHC analysis was performed using a Ventana XT automated stainer (Ventana Corporation, Tucson, AZ, USA) with antibodies against the following: MutL homolog 1 (MLH1, diluted 1:50, BD Biosciences, San Jose, CA, USA), MutS homolog 2 (MSH2, diluted 1:200, BD Biosciences), MutS homolog 6 (MSH6, diluted 1:100, Cell Marque, Rocklin, CA, USA), and PMS1 homolog 2 (PMS2, diluted 1:40, Cell Marque). Positive internal controls including stromal cells and lymphoid cells were confirmed, and the percentage of nuclear expression was measured.

### Statistical analysis

To calculate the diagnostic sensitivity and specificity of each MSI test, McNemar’s tests were performed. The sensitivity (%) of each MSI analysis method was calculated as follows: 100 × true positives/(true positives + false negatives), where true positives and false negatives were defined according to MSI status originally diagnosed by NCI method. The specificity (%) of PNA method was calculated as follows: 100 × true negatives/(false positives + true negatives), where true negatives and false positives were defined according to MSI status originally diagnosed by NCI method.

To determine the sample number required for this study, we performed non-inferiority test at a significance level of 0.05 and a statistical power of 80%. The average of positive predictive value (PPV) and negative predictive value (NPV) were obtained from 10 reference articles [11–20], where methods using microsatellite instability testing or real-time PCR were compared to immunohistochemistry for detecting mutations of MLH1, MSH2, MSH6, and PMS2 or other human genes. The average PPV and NPV were 91.1 and 93.6%, respectively, and the differences from lower margin of 95% confidence intervals (CI; δ, non-inferiority margin) were − 5.49% and − 4.3%, respectively. Based on reference articles, the sizes of MSI-H and MSS samples were calculated using the equation below, considering a 10% dropout rate.

$$ \mathrm{N}=\frac{{\left({Z}_{\alpha /2}+{Z}_{\beta}\right)}^2P\left(1-P\right)\ }{{\left(\delta -\left|P-{P}_0\right|\right)}^2} $$

*P* = average PPV and NPV values from reference articles; *P*_0_= expected PPV and NPV for this study (equivalence); *Z*_*α*/2_=1.96; *Z*_*β*_ = 0.842.

Based on the calculation above, we came to the conclusion that more than 68 MSI-H and 89 MSS CRC samples were required for this study, and performed MSI analysis in 76 MSI-H and 90 MSS CRC samples.

To analyze clinicopathological parameters, statistical analyses were performed using SPSS software, version 21.0.0.0 for Windows (IBM., Armonk, NY, USA). Mann-Whitney tests, Student’s t-tests, Fisher’s exact tests, or χ^2^ tests were used depending on the purpose; *P*-values < 0.05 were considered statistically significant.

## Results

### Determination of MSI status by three conventional MSI detection methods

We randomly collected 166 cases from 2263 CRCs that had undergone MSI status analysis with variable methods from January 2005 to December 2015. The NCI analysis results of the 166 cases were also collected. Seventy-six cases were previously diagnosed as MSI-H and 90 cases as MSS or MSI-L.

To test the performance of conventional MSI detection methods, we conducted MNR method with five quasi-monomorphic mononucleotide markers (Promega MSI Analysis System Kit) and IHC with four MMR proteins (MLH1, MSH2, MSH6, and PMS2). MSI analysis results determined by each method are summarized in Additional file 1: Table S1. Among the 76 MSI-H cases, 74 cases showed MSI-H in all three conventional MSI tests. Case no. 1 was diagnosed as MSI-H by NCI and IHC but was diagnosed as MSI-L by MNR. Case no. 20 was diagnosed as MSI-H by NCI and MNR, but no loss of protein expression was detected by IHC (Additional file 1: Table S1 and Additional file 2: Figure S1).

### Diagnostic sensitivity and specificity of three MSI test methods

To assess the clinical sensitivity and specificity of each MSI test, McNemar’s tests were performed by comparing the MSI analysis results of IHC, MNR, and PNA methods with the MSI status originally diagnosed by NCI method in the 166 CRCs (Table 1). The clinical sensitivity and specificity of PNA method were 100% (95% confidence interval (CI): 95.2–100%) and 100% (95% CI: 95.9–100%), respectively. On the other hand, the clinical sensitivity and specificity of MNR and IHC methods were 98.68% (95% CI: 92.9–99.8%) and 100% (95% CI: 95.9–100%), respectively.

**Table 1:** MSI analysis results of NCI, PNA, MNR, and IHC methods for 166 CRCs

Total Sample (*n* = 166)		MSI status		Sum
		MSI-H (*n* = 76)	MSS (*n* = 90)	
NCI method	MSI-H	76	0	76
	MSS	0	90	90
Sum		76	90	166
PNA method	MSI-H	76	0	76
(U-TOP™ MSI Detection Kit)	MSS	0	90	90
Sum		76	90	166
MNR method	MSI-H	75	0	75
(Promega MSI analysis system kit)	MSS	1	90	91
Sum		76	90	166
IHC method	MSI-H	75	0	75
	MSS	1	90	91
Sum		76	90	166

**Fig. 1:**
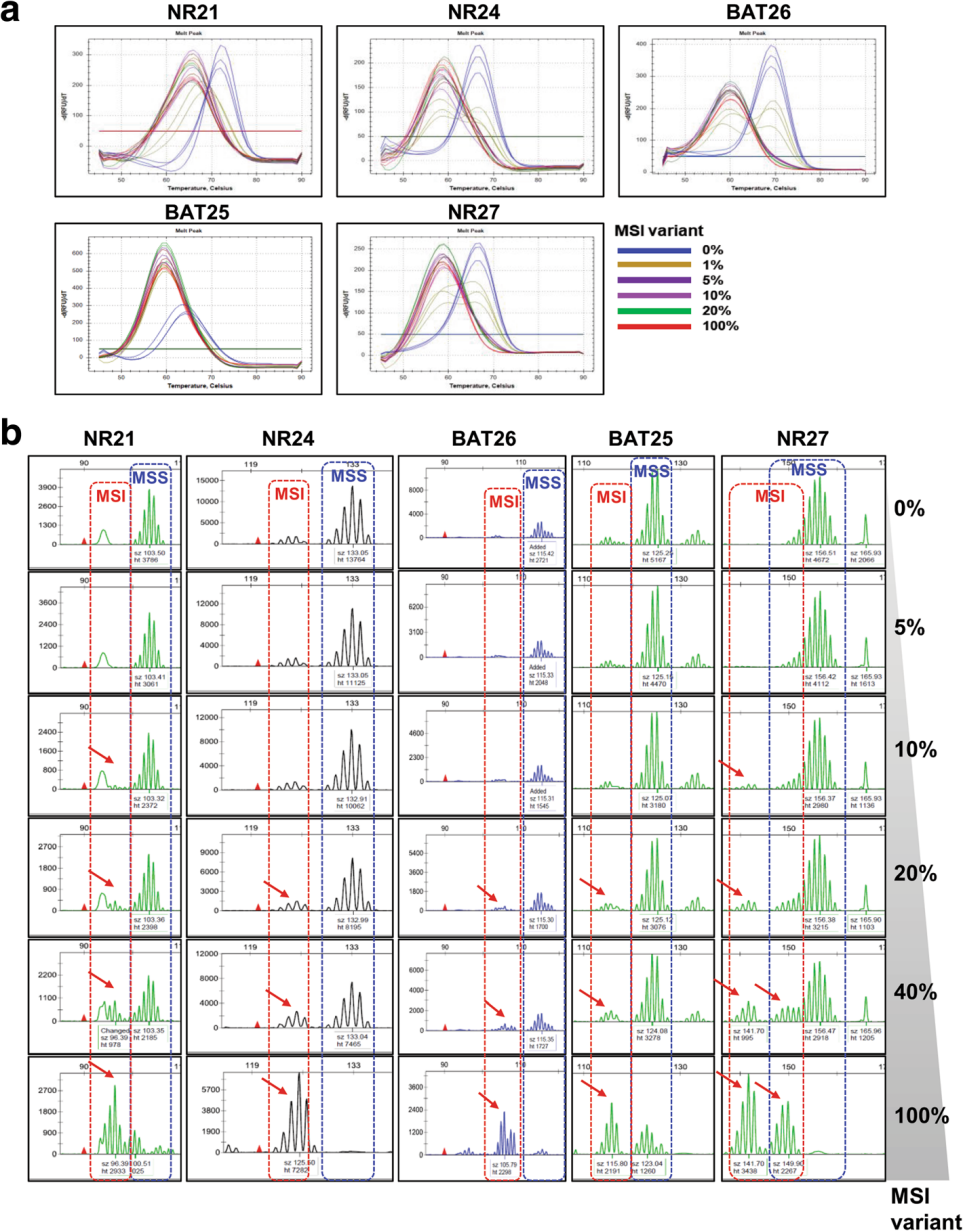
Maximum sensitivity evaluation of PNA and MNR method. The maximum sensitivity of PNA and MNR methods was evaluated using mixed samples of genomic DNA samples obtained from HeLa (MSS) and SNU-638 (MSI-H) cells. **a** PNA method was capable of detecting alteration in all five MSI marker genes in samples containing down to 5% MSI-H variant. **b** MNR method required at least 20% MSI-H variant in samples to detect alteration in all five MSI marker genes

**Fig. 2:**
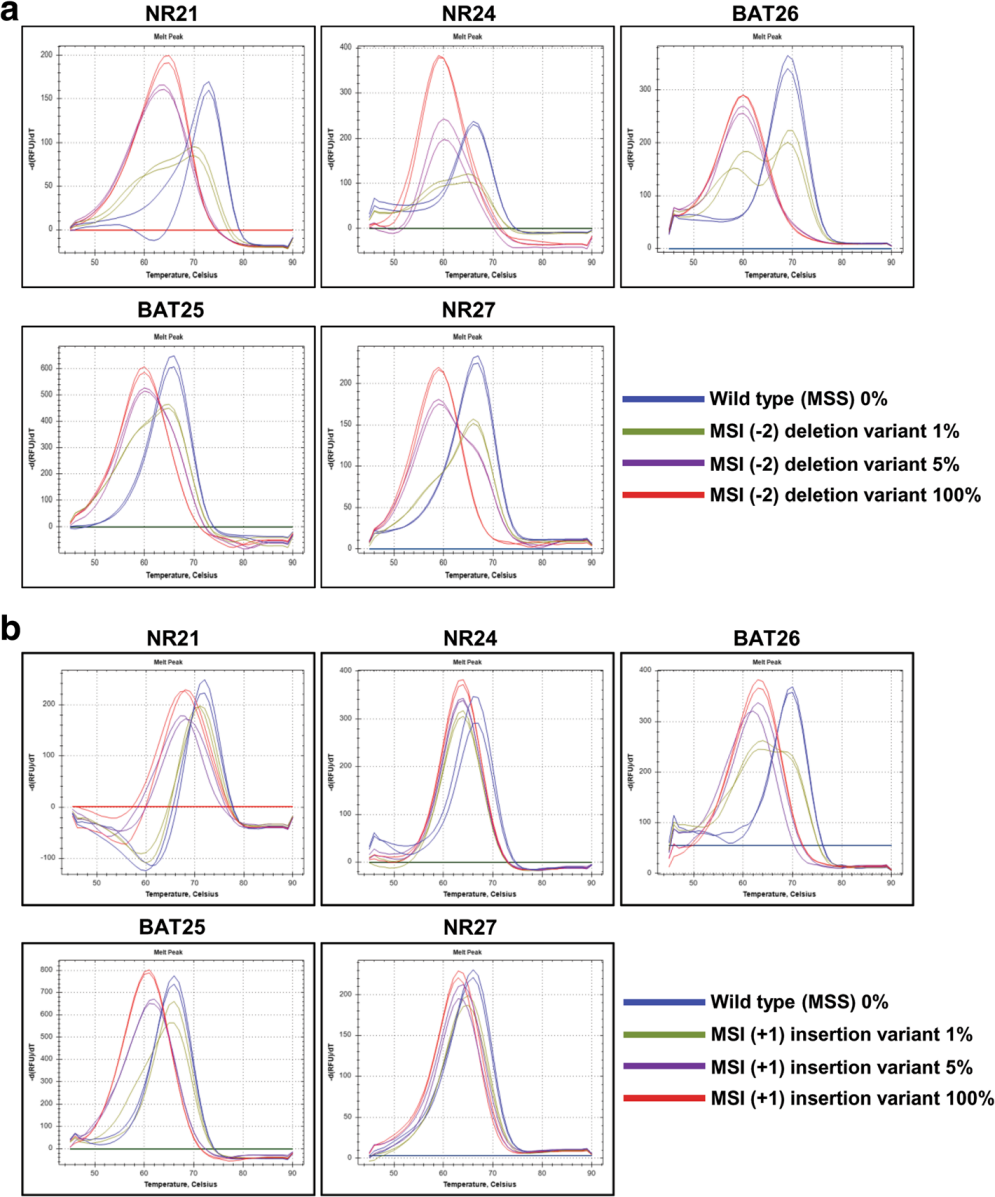
Evaluation of maximum sensitivity of PNA method using oligo targets containing minimal base pair alteration. **a** and **b** PNA analysis was performed using oligo samples containing different portions of MSI variants with two-base deletion or one-base insertion. PNA method clearly detected two-base deletion and one-base insertion in all five MSI markers in samples containing 5% or more MSI-H variant

Next, we evaluated the maximum sensitivity of the MNR and PNA methods, which used the same five MSI markers. To do so, mixed samples of gDNAs extracted from HeLa cells (microsatellite stable, MSS) and SNU-638 cells (MSI-H) were used, and MNR and PNA analyses were performed with different proportions (0, 1, 5, 10, 20, 40, and 100%) of MSI-H variant. MNR analysis showed that SNU-638 cells harbored 7-, 8-, 8-, 12-, and 10-base deletion mutations in *NR21*, *NR24*, *BAT25*, *BAT26*, and *NR27*, respectively. PNA method was capable of detecting alterations in all five MSI markers in the mixed samples, including down to 5% MSI-H variant, whereas MNR method required a sample containing up to 20% MSI-H variant to detect alterations in all five MSI markers. PNA method was further capable of detecting 1% MSI-H variant in NR21 and BAT25 markers (Fig. 1 and Additional file 3: Table S2). The repeatedly performed experiments using PNA method provided consistent and reproducible results, irrespective of the type of MSI markers and portion of MSI variants. To evaluate the qualitative performance of PNA method, we determined the minimum alteration number in each MSI marker that PNA method can detect, using artificially synthesized oligo targets containing one or two-base deletion mutations and one or two-base insertion mutations. The samples used in this experiment were composed of 100% MSI variant. Oligo targets containing insertion mutations were included because mononucleotide microsatellites often exhibit insertion mutations. The analysis result showed that PNA method clearly detected up to two-base deletion and one-base insertion mutations of the five MSI markers (Additional file 2: Figure S2). Then, we performed PNA analysis using titrated samples with MSI variants containing a two-base deletion or one-base insertion to evaluate the quantitative performance of PNA method. The analysis results showed that PNA method clearly detected a two-base deletion or one-base insertion of the five MSI markers in samples containing more than 5% MSI-H variants (Fig. 2).

### Determination of MSI status by the PNA probe-mediated melting point analysis

**Fig. 3:**
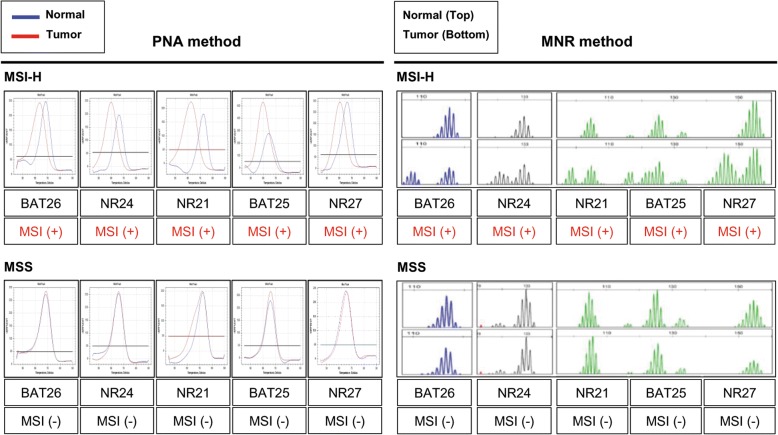
Analysis of MSI status in 166 CRCs and matched normal samples using PNA (left panel) and MNR method (right panel). Representative MSI analysis results of CRC samples determined as MSI-H or MSS are shown

MSI status was determined by PNA method, which was a recently developed real-time PCR based method. The results of PNA method showed sharp melting curves highly specific to each cancer sample as compared with paired normal samples (Fig. 3, left panel). MNR method also showed readable size differences between cancer and paired normal samples (Fig. 3, right panel). Samples with alterations in more than one MSI marker were determined as MSI-H, whereas samples with an alteration in a single MSI marker or no alteration were determined as MSI-L or MSS, respectively (Fig. 3 and Additional file 2: Figure S3). MSI-L and MSS were grouped together for statistical analysis based on a previous report of no significant clinicopathological or molecular differences between MSI-L and MSS CRCs [21]. MSI analysis using PNA method indicated that 76 samples were MSI-H and the remaining 90 were MSS, which was the same result as that of NCI analysis. A comparison of instability in each MSI marker between PNA and MNR method showed significant difference in *NR24,* with PNA having higher detection rates (Additional file 3: Table S3). We also note that one case (Case no. 1) was determined as MSI-L by MNR but was determined as MSI-H by NCI, IHC, and PNA methods (Additional file 1: Table S1).

### Clinicopathologic findings of sporadic and hereditary MSI-H CRCs

We observed that MSI-H and MSS CRCs showed distinctive clinicopathologic characteristics, as previously reported [2]. Compared with MSS CRCs, MSI-H CRCs were more frequently found in the right colon and showed larger and exophytic features. Elevated pre-operative carcinoembryonic antigen (CEA) level and advanced T stage were noted in MSS CRCs. Mucinous adenocarcinoma and signet ring cell carcinoma were observed more often in MSI-H CRCs than in MSS CRCs. Patients with MSI-H CRCs were younger on average than patients with MSS CRCs (Additional file 3: Table S4).

Among the 166 CRCs, 155 cases did not satisfy Amsterdam II and Revised Bethesda criteria, which allowed us to clinically define these cases as sporadic CRCs. Eleven cases showed clinical features of HNPCC as suggested by Amsterdam II or Revised Bethesda criteria. Analysis of these 11 clinically diagnosed HNPCC cases by NCI, MNR, IHC, and PNA methods showed that two cases were determined as MSS in all four tests, indicating that these two cases can be considered as familial colorectal cancer type X. In the remaining nine cases, MSI-H was determined by all four tests and mutations in *MLH1* or *MSH2* were detected (data not shown), which leads us to consider the nine cases are Lynch syndrome. Consequently, nine and 67 cases were classified as MSI-H CRCs associated with Lynch syndrome and sporadic MSI-H CRCs, respectively. Whereas most clinicopathologic characteristics of the nine cases with Lynch syndrome and 67 sporadic MSI-H CRC cases were similar, some parameters showed significant differences (Additional file 3: Table S5). Patients in the Lynch syndrome group were younger on average than patients in the sporadic CRC group. Also, mean tumor size was smaller in the Lynch syndrome group, perhaps because the features of hereditary CRCs, such as family history and early symptoms, may lead to early health check-ups and the detection of smaller tumors. There were no differences in other parameters such as MMR protein expression status, tumor location, pre-operative CEA level, gross type, histologic diagnosis, T stage, lymphovascular invasion, mucin formation, Crohn-like reaction, or tumor budding.

### Diagnostic value of IHC using antibodies against four MMR proteins to determine MSI status

Next, we performed IHC analysis of four MMR proteins in the 166 CRCs to determine MSI status. MSI-H was defined as the loss of expression of at least one MMR protein in more than 95% of tumor cells. By this criterion, we detected MSI-H in 75 out of 76 samples (98.7%). The percentage of nuclear expression of MMR proteins was also measured in all cases, and MSI status was further analyzed according to various cut-off values of loss of MMR protein expression (Additional file 3: Table S6). When we defined MMR deficit as a complete loss of nuclear staining, we observed 84.21% concordance with the originally diagnosed MSI status using two antibodies against MLH1 and MSH2, 73.68% concordance with two antibodies against PMS2 and MSH6, and 90.79% concordance with all four antibodies against MLH1, MSH2, PMS2, and MSH6. When we defined MMR deficit as < 1% nuclear staining, we observed 84.21% concordance with MLH1 and MSH2 antibodies, 89.47% concordance with PMS2 and MSH6 antibodies, and 96.05% with all four antibodies. When we defined MMR as < 5% nuclear staining, we observed 90.79% concordance with MLH1 and MSH2 antibodies and 98.68% concordance with PMS2 and MSH6 antibodies or all four antibodies. Overall, therefore, an IHC criterion of 95% was the best match to the original MSI diagnosis results.

## Discussion

The determination of MSI status is important because CRCs with MSI-H show distinguishing clinicopathologic characteristics and require optimized treatment [22, 23]. IHC for MMR proteins can effectively identify CRCs with a MSI-H subtype and provides indirect information about the affected MMR pathway. IHC is a relatively quick and simple assay for determining MSI status by evaluating the protein expression of four MMR genes: MLH1, MSH2, MSH6, and PMS2. MLH1 forms a functional complex with PMS2, and MSH2 forms a functional complex with MSH6. Because MLH1 is responsible for the stability of PMS2, the combined loss of PMS2 and MLH1 suggests that MLH1 is defective. Similarly, the combined loss of MSH2 and MSH6 suggests a defect within MSH2. Our finding that the co-loss of MLH1/PMS2 or MSH2/MSH6 predominantly occurred in MSI-H CRC samples further supports a relationship among MMR proteins.

We also observed variable MMR expression patterns such as loss of a single MMR marker, loss of MLH1 together with MSH2 and MSH6, loss of MSH6 together with MLH1 and PMS2, and loss of all four markers. Exclusive loss of PMS2 expression could be explained by a PMS2 or MLH1 mutation resulting in intact expression of MLH1 with abnormal function [24]. Loss of MSH6 together with MLH1 and PMS2 could occur because of a somatic mutation in MSH6 combined with MLH1 hypermethylation [25]. Rarely, a null pattern has reportedly been caused by a germline MSH2 mutation together with somatic MLH1 hypermethylation [26]. The interpretation of these rare MMR expression patterns is challenging, which limits the application of IHC analysis for MSI detection. Moreover, the interpretation of IHC results can be limited by the cut-off value. Although some studies suggest 5% or 10% cut-off values [27, 28], there is no consensus on the minimal percentage of nuclear staining that should be considered as intact expression. Because slight discordance between IHC and MSI molecular tests is rather natural [29], our results suggest that > 5% nuclear staining was the best match to MSI test results. The challenging interpretation of IHC results could be due to somatic missense mutations in sporadic CRCs that can reduce IHC staining without affecting MMR protein expression and thus not cause pathogenesis [30]. Tissue fixation status, somatic mosaicism, or other gene defects that also cause a MSI-H phenotype also could affect the interpretation of IHC results [28]. Indeed, there was one case (Case no. 20), determined as MSI-H by other molecular tests, that showed expression of all four MMR proteins. Therefore, we suggest that additional molecular tests of MSI status should be performed in cases with decreased expression of one or more MMR genes and/or clinicopathologic features related to the MSI-H subtype.

Several methods using amplicon melting analysis had been suggested for genotyping and mutation scanning. The primitive techniques had required a fluorescently, labeled primer, and been limited to the detection of mutations residing in the melting domain of the labeled primer. Taking advantage of a double-stranded DNA dye, Wittwer et al. reported another amplicon melting analysis method that did not require any labeled primers. This method was not limited by the requirement that sequence variants have to be in the same melting domain [31]. Likewise, real-time PCR based methods have been developed for the analysis of various DNA alterations. Here, we evaluated the performance of three MSI detection molecular tests and found that PNA method can be used as a time- and cost-efficient molecular test for MSI diagnosis. Instead of using a set of five marker genes (two mononucleotide repeats and three dinucleotide repeats) recommended by the NCI, PNA method employs a panel of five marker genes containing quasi-monomorphic mononucleotide repeats proposed by Buhard et al. [32]. Many previous studies have shown that quasi-monomorphic mononucleotide repeats are far more sensitive than dinucleotide repeats in detecting MSI [33–35]. PNA method adopts a PNA-based real-time PCR sensing strategy that can be performed by a conventional real-time PCR machine and requires only a small amount of sample. We demonstrate that PNA method was capable of detecting a very small proportion of a mutant gene variant (5%) in a mixed sample of wild-type and mutant gDNAs, which complies with the College of American Pathologists guideline that molecular tests should be capable of detecting mutation in specimens with > 5% tumor cell population [36]. PNA probes enable strong amplification of mutant and weak amplification of wild type allele in a sample containing wild type and mutant alleles, which guarantees sensitive detection of mutant variants without an internal positive control. Since most clinical samples contain both normal and mutant variants, PNA method shows higher utilization over previous melting point analysis methods. As shown in the MNR analysis results of *NR24* and *BAT25* in samples containing 20% MSI-H variants (Fig. 1b), interpretation of data from MNR could be highly confusing and subjective. This might be due to artifacts in capillary electrophoresis that appear as smaller, split, and stutter peaks. Challenging interpretation of MNR analysis results likely reduces data reproducibility. On the other hand, PNA method provides data consisting of clear and sharp melting peaks detected at a specific melting temperature, which facilitates interpretation of data for researchers. There are some demerits also in the PNA method. The procedure for PNA method takes relatively longer than a general real-time PCR protocol for accuracy reasons (requiring ~ 4.5 h), and PNA method cannot distinguish homozygotes from heterozygotes, which might not be a critical disadvantage for this method due to using quasi-monomorphic markers. In terms of cost-efficiency, the cost for running PNA method is about three-fifths of that for PCR fragment analysis-based MSI tests (MNR and NCI methods). Moreover, PNA method can be performed in a general real-time PCR machine, which costs only one-fourths of a sequencer required for PCR fragment analysis. Overall, PNA method has some advantages over MNR and NCI methods.

The determination of MSI status is critical for the intense lifelong screening of patients with Lynch syndrome and the appropriate treatment of patients with sporadic CRCs. Amsterdam criteria (revised to Amsterdam II criteria in 1998) and revised Bethesda criteria are used to identify HNPCCs, which could lead to the misdiagnosis of some CRC patients with Lynch syndrome. Therefore, it is highly recommended that every diagnosed CRC patient undergo Lynch syndrome screening by IHC and/or molecular tests. However, Lynch syndrome screening for every colon cancer patient is not cost-effective. We therefore propose two possible screening algorithms, both of which employ screening for MSI using IHC for PMS2 and MSH6 based on our finding that the detection rate of MSI using these two markers was the same as that using all four markers when the cut-off value was ≤5% nuclear expression (Additional file 3: Table S6). In both algorithms, CRC patients are divided into two groups depending on clinical criteria such as Amsterdam II or Revised Bethesda criteria. In the first algorithm, MSI screening is initially performed by IHC using PMS2 and MSH6 markers, which reduces the cost by half compared to applying IHC analysis in four MMR markers. Next, a cost- and time-efficient tool, such as PNA, is applied to determine MSI presence in CRCs, which also reduces the cost by three-fifths and time by half compared to using conventional molecular tests (Additional file 2: Figure S4). In the second algorithm, MSI screening is initially performed using PNA and subsequent IHC analysis is performed to identify suspected gene(s), which could be similarly time- and cost-efficient as the first algorithm (Additional file 2: Figure S5). However, a molecular test for MSI analysis should be carefully selected since, test results from independent methods that even use the same MSI markers sometimes show discrepancy. In our cohort, there was a sample (Case no. 1) that was determined as MSI-L by MNR method, but as MSI-H by PNA method. In this case, comparing two results is possible, but deciding which is correct can be difficult.

## Conclusions

In this study, we have evaluated the MSI detection performance of a recently developed PNA method, conventional molecular tests, and immunohistochemistry. Based on our findings, we suggest that PNA method could be used as a simple alternative to existing molecular tests.

## Additional files

Additional file 1: **Table S1.** MSI status as determined by NCI, MNR, IHC, and PNA methods for 166 CRCs. (PDF 514 kb)

Additional file 2: **Figure S1.** Case no. 20 was diagnosed as MSI-H by NCI, MNR, and PNA methods but no loss of nuclear expression was detected using IHC for MMR proteins. **Figure S2.** Determination of minimal base alteration that can be detected by PNA method. (a and b) PNA analysis was performed using artificially synthesized MSI variants containing − 1 or − 2 deletion mutations and + 1 or + 2 insertion mutations. **Figure S3.** Representative MSI analysis results of CRC samples determined as MSI-L by PNA (left panel) and MNR method (right panel). **Figure S4.** Type 1 algorithm for MSI screening of sporadic and hereditary CRC patients by IHC and subsequent molecular tests. **Figure S5.** Type 2 algorithm for MSI screening of sporadic and hereditary CRC patients by molecular tests and subsequent IHC. (PDF 447 kb)

Additional file 3: **Table S2.** Sensitivity of MNR and PNA method. **Table S3.** MSI-H detection rate for each marker used in PNA and MNR method. **Table S4.** Clinicopathologic characteristics of 76 MSI-H CRC patients and 90 MSS CRC patients. **Table S5.** Clinicopathologic characteristics of patients with MSI-H CRCs associated with sporadic conditions or Lynch syndrome. **Table S6.** IHC-mediated MSI-H detection rate depending on the combination of markers and variable cut-off values for loss of MMR protein expression. (PDF 342 kb)

## Abbreviations

CRC: Colorectal carcinoma
FFPE: Formalin-fixed paraffin-embedded
HNPCC: Hereditary non-polyposis colorectal cancer
IHC: Immunohistochemistry
MMR: Mismatch repair
MSI-H: High microsatellite instability
MSI-L: Low microsatellite instability
MSS: Microsatellite stable
PCR: Polymerase chain reaction
PNA: Peptide nucleotide acid
