# Supplementary material for: Microsatellite instability test using peptide nucleic acid probe-mediated melting point analysis: a comparison study
Source: BMC Cancer. 2018 Dec 4;18:1218. doi: 10.1186/s12885-018-5127-6 (PMC6280403; doi:10.1186/s12885-018-5127-6)
Supplement: Supplementary file 2 — Figure S1. Case no. 20 was diagnosed as MSI-H by NCI, MNR, and PNA methods but no loss of nuclear expression was detected using IHC for MMR proteins. Figure S2. Determination of minimal base alteration that can be detected by PNA method. (a and b) PNA analysis was performed using artificially synthesized MSI variants containing − 1 or − 2 deletion mutations and + 1 or + 2 insertion mutations. Figure S3. Representative MSI analysis results of CRC samples determined as MSI-L by PNA (left panel) and MNR method (right panel). Figure S4. Type 1 algorithm for MSI screening of sporadic and hereditary CRC patients by IHC and subsequent molecular tests. Figure S5. Type 2 algorithm for MSI screening of sporadic and hereditary CRC patients by molecular tests and subsequent IHC. (PDF 447 kb) [file 12885_2018_5127_MOESM2_ESM.pdf]

Supplementary Figure S1

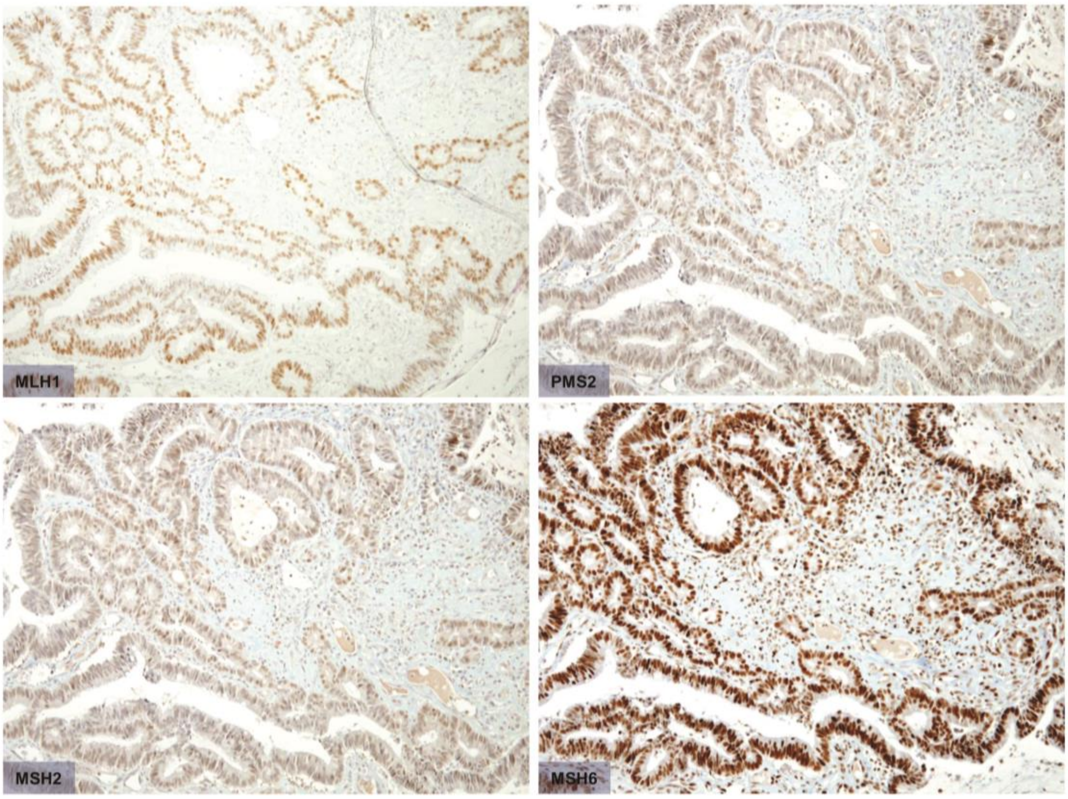

Supplementary Fig. S1 Case no. 20 was diagnosed as MSI-H by NCI, MNR, and PNA methods but no loss of nuclear expression was detected using IHC for MMR proteins.

Supplementary Figure S2

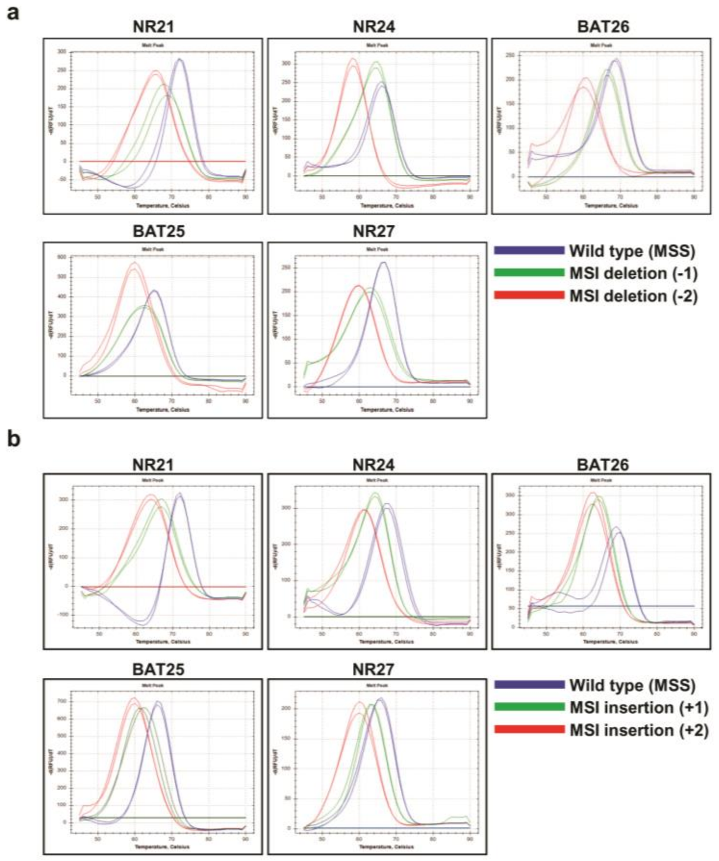

Supplementary Fig. S2 Determination of minimal base alteration that can be detected by PNA method. (**a and b**) PNA analysis was performed using artificially synthesized MSI variants containing -1 or -2 deletion mutations and +1 or +2 insertion mutations.

Supplementary Figure S3

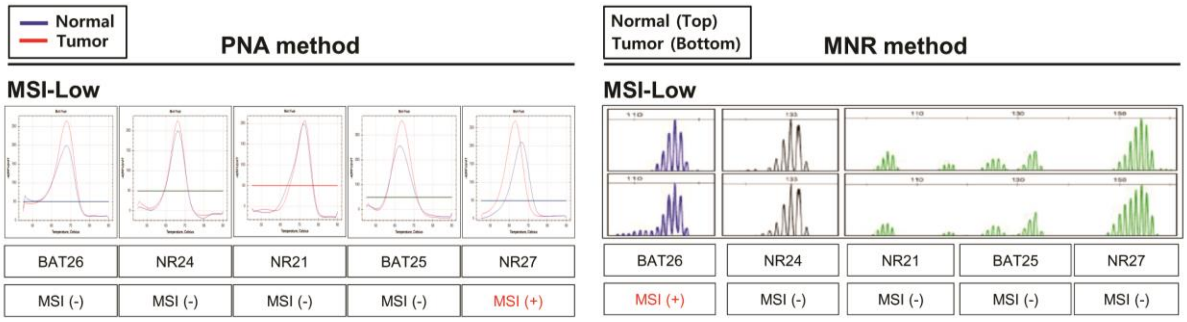

Supplementary Fig. S3 Representative MSI analysis results of CRC samples determined as MSI-L by PNA (left panel) and MNR method (right panel).

Supplementary Figure S4

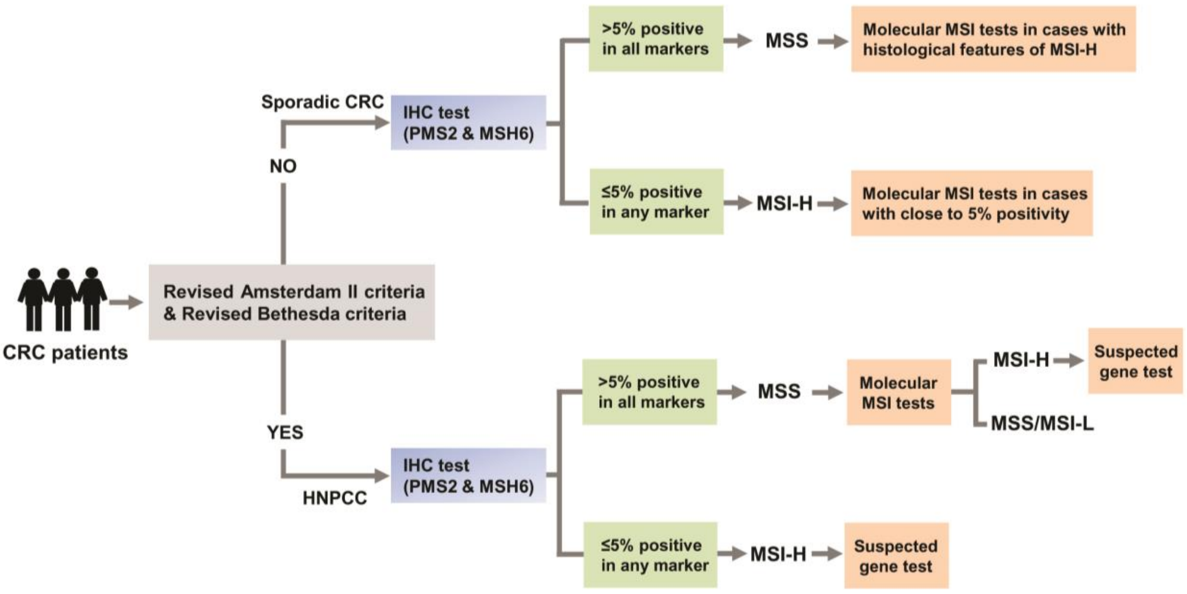

Supplementary Fig. S4 Type 1 algorithm for MSI screening of sporadic and hereditary CRC patients by IHC and subsequent molecular tests.

Supplementary Figure S5

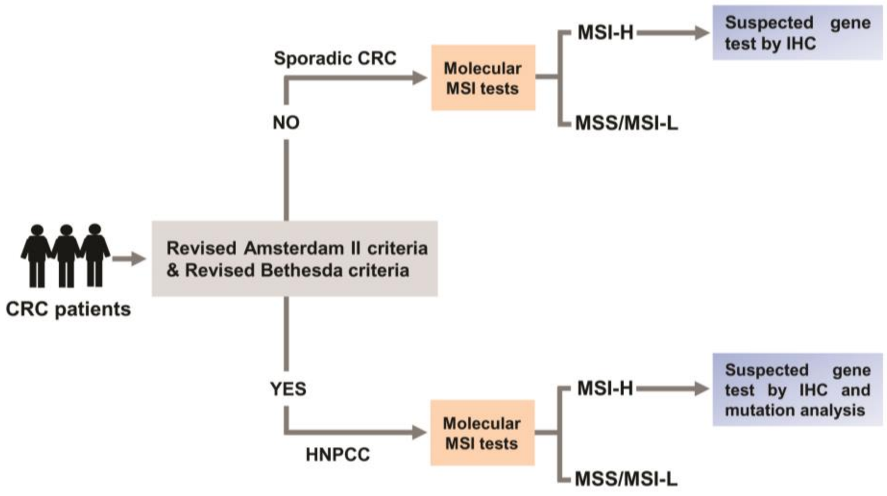

Supplementary Fig. S5 Type 2 algorithm for MSI screening of sporadic and hereditary CRC patients by molecular tests and subsequent IHC.
