# Supplementary material for: Microsatellite instability test using peptide nucleic acid probe-mediated melting point analysis: a comparison study
Source: BMC Cancer. 2018 Dec 4;18:1218. doi: 10.1186/s12885-018-5127-6 (PMC6280403; doi:10.1186/s12885-018-5127-6)
Supplement: Supplementary file 1 — Table S1. MSI status as determined by NCI, MNR, IHC, and PNA methods for 166 CRCs. (PDF 514 kb) [file 12885_2018_5127_MOESM1_ESM.pdf]

Supplementary Table S1 MSI status as determined by NCI, MNR, IHC, and PNA methods for 166 CRCs

Sample	NCI method						MNR method						Immunohistochemistry method																			PNA method					
	Marker					MSI status	Marker					MSI status	Nuclear expression percentage				Cut off value: complete loss				MSI status	Cut off value: ≥99% loss				MSI status	Cut off value: ≥95%				MSI status	Marker					MSI status
	BAT26	*D5S346*	*BAT25*	*D17S250*	*D2S123*		*NR21*	*NR24*	*BAT26*	*BAT25*	*NR27*		MLH1	PMS2	MSH2	MSH6	MLH1	PMS2	MSH2	MSH6		MLH1	PMS2	MSH2	MSH6		MLH1	PMS2	MSH2	MSH6		*NR21*	*NR24*	*BAT26*	*BAT25*	*NR27*	
1	+	+	+	+	+	MSI-H	—	—	—	—	+	MSI-L	0	0	80	100	Loss	Loss	No loss	No loss	MSI-H	Loss	Loss	No loss	No loss	MSI-H	Loss	Loss	No loss	No loss	MSI-H	+	+	+	+	+	MSI-H
2	+	+	+	+	+	MSI-H	—	+	—	+	+	MSI-H	0	0	70	95	Loss	Loss	No loss	No loss	MSI-H	Loss	Loss	No loss	No loss	MSI-H	Loss	Loss	No loss	No loss	MSI-H	+	+	+	+	+	MSI-H
3	+	+	+	+	+	MSI-H	+	+	—	+	+	MSI-H	0	0	40	30	Loss	Loss	No loss	No loss	MSI-H	Loss	Loss	No loss	No loss	MSI-H	Loss	Loss	No loss	No loss	MSI-H	+	+	+	+	+	MSI-H
4	+	+	+	+	+	MSI-H	+	+	—	+	+	MSI-H	80	95	0	0	No loss	No loss	Loss	Loss	MSI-H	No loss	No loss	Loss	Loss	MSI-H	No loss	No loss	Loss	Loss	MSI-H	+	+	+	+	+	MSI-H
5	+	+	+	—	—	MSI-H	+	+	+	+	—	MSI-H	100	100	90	0	No loss	No loss	No loss	Loss	MSI-H	No loss	No loss	No loss	Loss	MSI-H	No loss	No loss	No loss	Loss	MSI-H	+	+	+	+	+	MSI-H
6	+	+	+	+	+	MSI-H	+	+	+	+	+	MSI-H	90	100	0	4	No loss	No loss	Loss	No loss	MSI-H	No loss	No loss	Loss	No loss	MSI-H	No loss	No loss	Loss	Loss	MSI-H	+	+	—	+	+	MSI-H
7	+	+	+	+	+	MSI-H	+	+	—	+	+	MSI-H	0	0	60	70	Loss	Loss	No loss	No loss	MSI-H	Loss	Loss	No loss	No loss	MSI-H	Loss	Loss	No loss	No loss	MSI-H	+	+	+	+	+	MSI-H
8	+	+	+	+	+	MSI-H	+	+	—	+	+	MSI-H	0	0	90	95	Loss	Loss	No loss	No loss	MSI-H	Loss	Loss	No loss	No loss	MSI-H	Loss	Loss	No loss	No loss	MSI-H	+	+	+	+	+	MSI-H
9	+	+	+	+	+	MSI-H	+	+	+	+	+	MSI-H	0	0	90	95	Loss	Loss	No loss	No loss	MSI-H	Loss	Loss	No loss	No loss	MSI-H	Loss	Loss	No loss	No loss	MSI-H	+	+	—	+	+	MSI-H
10	+	—	+	+	+	MSI-H	+	+	+	+	+	MSI-H	98	100	0	<1	No loss	No loss	Loss	No loss	MSI-H	No loss	No loss	Loss	Loss	MSI-H	No loss	No loss	Loss	Loss	MSI-H	+	+	+	+	+	MSI-H
11	+	+	+	+	+	MSI-H	+	+	+	+	+	MSI-H	0	0	90	95	Loss	Loss	No loss	No loss	MSI-H	Loss	Loss	No loss	No loss	MSI-H	Loss	Loss	No loss	No loss	MSI-H	+	+	+	+	+	MSI-H
12	+	—	+	+	+	MSI-H	+	+	+	+	+	MSI-H	99	100	5	<1	No loss	No loss	No loss	No loss	MSS	No loss	No loss	No loss	Loss	MSI-H	No loss	No loss	Loss	Loss	MSI-H	+	+	+	—	+	MSI-H
13	+	+	+	+	+	MSI-H	+	+	—	+	+	MSI-H	0	0	90	80	Loss	Loss	No loss	No loss	MSI-H	Loss	Loss	No loss	No loss	MSI-H	Loss	Loss	No loss	No loss	MSI-H	+	+	+	+	+	MSI-H
14	+	+	+	—	+	MSI-H	+	+	+	+	+	MSI-H	100	95	50	0	No loss	No loss	No loss	Loss	MSI-H	No loss	No loss	No loss	Loss	MSI-H	No loss	No loss	No loss	Loss	MSI-H	+	+	+	+	+	MSI-H
15	+	+	+	+	+	MSI-H	+	+	+	+	+	MSI-H	0	0	95	95	Loss	Loss	No loss	No loss	MSI-H	Loss	Loss	No loss	No loss	MSI-H	Loss	Loss	No loss	No loss	MSI-H	+	+	+	+	+	MSI-H
16	+	+	+	+	+	MSI-H	+	+	—	+	+	MSI-H	0	0	90	90	Loss	Loss	No loss	No loss	MSI-H	Loss	Loss	No loss	No loss	MSI-H	Loss	Loss	No loss	No loss	MSI-H	+	+	+	+	+	MSI-H
17	+	+	+	+	+	MSI-H	+	+	+	+	+	MSI-H	0	0	85	95	Loss	Loss	No loss	No loss	MSI-H	Loss	Loss	No loss	No loss	MSI-H	Loss	Loss	No loss	No loss	MSI-H	+	+	+	+	+	MSI-H
18	+	+	+	+	+	MSI-H	+	+	+	+	+	MSI-H	0	<1	70	95	Loss	No loss	No loss	No loss	MSI-H	Loss	Loss	No loss	No loss	MSI-H	Loss	Loss	No loss	No loss	MSI-H	+	+	+	+	+	MSI-H
19	+	+	+	+	+	MSI-H	+	—	+	+	+	MSI-H	100	95	0	5	No loss	No loss	Loss	No loss	MSI-H	No loss	No loss	Loss	No loss	MSI-H	No loss	No loss	Loss	Loss	MSI-H	+	+	+	+	+	MSI-H
20	+	+	+	+	+	MSI-H	+	+	+	+	+	MSI-H	100	95	90	85	No loss	No loss	No loss	No loss	MSS	No loss	No loss	No loss	No loss	MSS	No loss	No loss	No loss	No loss	MSS	+	+	—	+	+	MSI-H
21	+	+	+	+	+	MSI-H	+	+	+	+	+	MSI-H	90	95	90	3	No loss	No loss	No loss	No loss	MSS	No loss	No loss	No loss	No loss	MSS	No loss	No loss	No loss	Loss	MSI-H	+	+	+	+	+	MSI-H
22	+	+	+	+	+	MSI-H	+	+	+	+	+	MSI-H	0	0	100	95	Loss	Loss	No loss	No loss	MSI-H	Loss	Loss	No loss	No loss	MSI-H	Loss	Loss	No loss	No loss	MSI-H	+	+	+	+	+	MSI-H
23	+	+	+	+	+	MSI-H	—	+	+	—	+	MSI-H	0	0	4	5	Loss	Loss	No loss	No loss	MSI-H	Loss	Loss	No loss	No loss	MSI-H	Loss	Loss	Loss	Loss	MSI-H	+	+	+	+	+	MSI-H
24	+	+	+	+	+	MSI-H	+	+	+	+	+	MSI-H	0	0	95	100	Loss	Loss	No loss	No loss	MSI-H	Loss	Loss	No loss	No loss	MSI-H	Loss	Loss	No loss	No loss	MSI-H	+	+	+	+	+	MSI-H
25	+	—	+	+	+	MSI-H	+	+	+	+	+	MSI-H	90	95	0	<1	No loss	No loss	Loss	No loss	MSI-H	No loss	No loss	Loss	Loss	MSI-H	No loss	No loss	Loss	Loss	MSI-H	+	+	—	+	+	MSI-H
26	+	—	+	+	+	MSI-H	+	+	+	+	+	MSI-H	99	100	0	<1	No loss	No loss	Loss	No loss	MSI-H	No loss	No loss	Loss	Loss	MSI-H	No loss	No loss	Loss	Loss	MSI-H	+	+	+	+	+	MSI-H
27	+	+	+	+	+	MSI-H	+	—	+	—	—	MSI-H	0	0	100	100	Loss	Loss	No loss	No loss	MSI-H	Loss	Loss	No loss	No loss	MSI-H	Loss	Loss	No loss	No loss	MSI-H	+	—	+	—	+	MSI-H
28	+	+	+	+	—	MSI-H	+	+	+	+	+	MSI-H	0	0	90	95	Loss	Loss	No loss	No loss	MSI-H	Loss	Loss	No loss	No loss	MSI-H	Loss	Loss	No loss	No loss	MSI-H	+	+	+	+	+	MSI-H
29	+	—	+	+	+	MSI-H	+	+	+	+	+	MSI-H	0	0	60	60	Loss	Loss	No loss	No loss	MSI-H	Loss	Loss	No loss	No loss	MSI-H	Loss	Loss	No loss	No loss	MSI-H	+	+	+	+	+	MSI-H
30	+	+	+	+	+	MSI-H	+	+	+	+	+	MSI-H	0	0	90	95	Loss	Loss	No loss	No loss	MSI-H	Loss	Loss	No loss	No loss	MSI-H	Loss	Loss	No loss	No loss	MSI-H	+	+	+	+	+	MSI-H
31	+	+	+	+	+	MSI-H	+	+	+	+	+	MSI-H	0	35	0	0	Loss	No loss	Loss	Loss	MSI-H	Loss	No loss	Loss	Loss	MSI-H	No loss	No loss	Loss	Loss	MSI-H	+	+	+	+	—	MSI-H
32	+	+	+	+	+	MSI-H	+	+	+	+	+	MSI-H	0	0	95	95	Loss	Loss	No loss	No loss	MSI-H	Loss	Loss	No loss	No loss	MSI-H	Loss	Loss	No loss	No loss	MSI-H	+	+	+	+	+	MSI-H
33	+	—	+	+	+	MSI-H	+	+	+	+	+	MSI-H	100	100	0	<1	No loss	No loss	Loss	No loss	MSI-H	No loss	No loss	Loss	Loss	MSI-H	No loss	No loss	Loss	Loss	MSI-H	+	+	+	+	+	MSI-H
34	+	+	+	+	+	MSI-H	+	+	+	+	+	MSI-H	0	0	100	95	Loss	Loss	No loss	No loss	MSI-H	Loss	Loss	No loss	No loss	MSI-H	Loss	Loss	No loss	No loss	MSI-H	+	+	+	+	+	MSI-H
35	+	—	+	—	+	MSI-H	+	+	+	+	+	MSI-H	0	0	90	10	Loss	Loss	No loss	No loss	MSI-H	Loss	Loss	No loss	No loss	MSI-H	Loss	Loss	No loss	No loss	MSI-H	+	+	+	+	+	MSI-H
36	+	+	+	+	+	MSI-H	+	—	+	+	+	MSI-H	70	90	0	2	No loss	No loss	Loss	No loss	MSI-H	No loss	No loss	Loss	No loss	MSI-H	No loss	No loss	Loss	Loss	MSI-H	+	+	+	+	+	MSI-H
37	+	+	+	+	+	MSI-H	+	+	+	+	+	MSI-H	0	<1	60	70	Loss	No loss	No loss	No loss	MSI-H	Loss	Loss	No loss	No loss	MSI-H	Loss	Loss	No loss	No loss	MSI-H	+	+	+	+	+	MSI-H
38	+	+	+	+	+	MSI-H	+	+	+	+	+	MSI-H	100	100	95	0	No loss	No loss	No loss	Loss	MSI-H	No loss	No loss	No loss	Loss	MSI-H	No loss	No loss	No loss	Loss	MSI-H	+	+	+	+	+	MSI-H
39	+	—	+	+	—	MSI-H	+	+	—	+	+	MSI-H	0	0	100	100	Loss	Loss	No loss	No loss	MSI-H	Loss	Loss	No loss	No loss	MSI-H	Loss	Loss	No loss	No loss	MSI-H	+	+	+	+	+	MSI-H
40	+	+	+	+	+	MSI-H	+	+	+	+	+	MSI-H	0	0	95	100	Loss	Loss	No loss	No loss	MSI-H	Loss	Loss	No loss	No loss	MSI-H	Loss	Loss	No loss	No loss	MSI-H	+	+	+	+	+	MSI-H
41	+	+	+	+	+	MSI-H	+	+	+	+	+	MSI-H	0	0	99	95	Loss	Loss	No loss	No loss	MSI-H	Loss	Loss	No loss	No loss	MSI-H	Loss	Loss	No loss	No loss	MSI-H	+	+	+	+	+	MSI-H
42	+	—	+	+	+	MSI-H	+	+	+	+	+	MSI-H	90	90	90	<1	No loss	No loss	No loss	No loss	MSS	No loss	No loss	No loss	Loss	MSI-H	No loss	No loss	No loss	Loss	MSI-H	+	+	+	+	+	MSI-H
43	+	+	+	—	+	MSI-H	+	+	+	+	+	MSI-H	90	70	5	<1	No loss	No loss	No loss	No loss	MSS	No loss	No loss	No loss	Loss	MSI-H	No loss	No loss	Loss	Loss	MSI-H	+	+	+	+	+	MSI-H
44	+	+	+	+	+	MSI-H	+	+	+	+	+	MSI-H	0	0	100	99	Loss	Loss	No loss	No loss	MSI-H	Loss	Loss	No loss	No loss	MSI-H	Loss	Loss	No loss	No loss	MSI-H	+	+	+	+	+	MSI-H
45	+	+	+	+	+	MSI-H	+	—	+	+	+	MSI-H	90	70	0	5	No loss	No loss	Loss	No loss	MSI-H	No loss	No loss	Loss	No loss	MSI-H	No loss	No loss	Loss	Loss	MSI-H	+	+	+	+	+	MSI-H
46	+	+	+	+	+	MSI-H	+	+	+	+	+	MSI-H	0	0	95	95	Loss	Loss	No loss	No loss	MSI-H	Loss	Loss	No loss	No loss	MSI-H	Loss	Loss	No loss	No loss	MSI-H	+	+	+	+	+	MSI-H
47	+	+	+	+	+	MSI-H	+	+	+	+	+	MSI-H	0	0	80	95	Loss	Loss	No loss	No loss	MSI-H	Loss	Loss	No loss	No loss	MSI-H	Loss	Loss	No loss	No loss	MSI-H	+	+	+	+	+	MSI-H
48	+	+	+	+	+	MSI-H	+	—	+	+	+	MSI-H	80	90	0	<1	No loss	No loss	Loss	No loss	MSI-H	No loss	No loss	Loss	Loss	MSI-H	No loss	No loss	Loss	Loss	MSI-H	+	+	+	+	+	MSI-H
49	+	+	+	—	+	MSI-H	+	—	+	+	+	MSI-H	0	<1	100	100	Loss	No loss	No loss	No loss	MSI-H	Loss	Loss	No loss	No loss	MSI-H	Loss	Loss	No loss	No loss	MSI-H	+	—	+	+	+	MSI-H
50	+	+	+	+	+	MSI-H	+	+	+	+	+	MSI-H	0	0	95	80	Loss	Loss	No loss	No loss	MSI-H	Loss	Loss	No loss	No loss	MSI-H	Loss	Loss	No loss	No loss	MSI-H	+	+	+	+	+	MSI-H
51	+	+	+	+	+	MSI-H	+	+	+	+	+	MSI-H	0	0	95	70	Loss	Loss	No loss	No loss	MSI-H	Loss	Loss	No loss	No loss	MSI-H	Loss	Loss	No loss	No loss	MSI-H	+	+	+	+	+	MSI-H
52	+	+	+	+	+	MSI-H	+	+	+	+	+	MSI-H	60	0	70	80	No loss	Loss	No loss	No loss	MSI-H	No loss	Loss	No loss	No loss	MSI-H	No loss	Loss	No loss	No loss	MSI-H	+	+	+	+	+	MSI-H
53	—	+	+	+	+	MSI-H	+	+	—	+	+	MSI-H	90	100	0	0	No loss	No loss	Loss	Loss	MSI-H	No loss	No loss	Loss	Loss	MSI-H	No loss	No loss	Loss	Loss	MSI-H	+	+	—	+	+	MSI-H
54	+	+	+	+	+	MSI-H	—	+	+	—	+	MSI-H	95	95	0	<1	No loss	No loss	Loss	No loss	MSI-H	No loss	No loss	Loss	No loss	MSI-H	No loss	No loss	Loss	Loss	MSI-H	—	+	+	—	+	MSI-H
55	+	+	+	+	+	MSI-H	+	+	—	+	+	MSI-H	0	0	95	100	Loss	Loss	No loss	No loss	MSI-H	Loss	Loss	No loss	No loss	MSI-H	Loss	Loss	No loss	No loss	MSI-H	+	+	+	+	+	MSI-H
56	+	+	+	+	—	MSI-H	+	—	+	+	+	MSI-H	0	0	100	100	Loss	Loss	No loss	No loss	MSI-H	Loss	Loss	No loss	No loss	MSI-H	Loss	Loss	No loss	No loss	MSI-H	—	—	+	+	+	MSI-H
57	+	+	+	+	+	MSI-H	+	—	+	+	+	MSI-H	0	0	80	70	Loss	Loss	No loss	No loss	MSI-H	Loss	Loss	No loss	No loss	MSI-H	Loss	Loss	No loss	No loss	MSI-H	+	+	+	+	+	MSI-H
58	+	+	+	+	—	MSI-H	+	—	+	+	+	MSI-H	80	95	0	0	No loss	No loss	Loss	Loss	MSI-H	No loss	No loss	Loss	Loss	MSI-H	No loss	No loss	Loss	Loss	MSI-H	+	+	+	+	+	MSI-H
59	+	+	+	+	+	MSI-H	+	—	+	+	+	MSI-H	0	0	95	95	Loss	Loss	No loss	No loss	MSI-H	Loss	Loss	No loss	No loss	MSI-H	Loss	Loss	No loss	No loss	MSI-H	+	+	+	+	+	MSI-H
60	+	+	+	+	+	MSI-H	+	+	+	+	+	MSI-H	0	0	90	80	Loss	Loss	No loss	No loss	MSI-H	Loss	Loss	No loss	No loss	MSI-H	Loss	Loss	No loss	No loss	MSI-H	+	+	+	+	+	MSI-H
61	+	+	+	+	+	MSI-H	+	+	+	+	+	MSI-H	0	0	100	95	Loss	Loss	No loss	No loss	MSI-H	Loss	Loss	No loss	No loss	MSI-H	Loss	Loss	No loss	No loss	MSI-H	+	+	+	+	+	MSI-H
62	+	+	+	+	+	MSI-H	+	+	+	+	+	MSI-H	0	0	100	100	Loss	Loss	No loss	No loss	MSI-H	Loss	Loss	No loss	No loss	MSI-H	Loss	Loss	No loss	No loss	MSI-H	+	+	+	+	+	MSI-H
63	+	+	+	+	+	MSI-H	+	+	—	+	+	MSI-H	0	0	10	4	Loss	Loss	No loss	No loss	MSI-H	Loss	Loss	No loss	No loss	MSI-H	Loss	Loss	No loss	Loss	MSI-H	+	+	+	+	+	MSI-H
64	+	+	+	+	+	MSI-H	+	+	—	+	+	MSI-H	0	0	100	100	Loss	Loss	No loss	No loss	MSI-H	Loss	Loss	No loss	No loss	MSI-H	Loss	Loss	No loss	No loss	MSI-H	+	+	+	+	+	MSI-H
65	+	+	+	+	+	MSI-H	+	+	+	+	+	MSI-H	0	0	80	60	Loss	Loss	No loss	No loss	MSI-H	Loss	Loss	No loss	No loss	MSI-H	Loss	Loss	No loss	No loss	MSI-H	+	+	+	+	+	MSI-H
66	+	+	+	+	+	MSI-H	+	+	+	+	+	MSI-H	3	0	95	95	No loss	Loss	No loss	No loss	MSI-H	No loss	Loss	No loss	No loss	MSI-H	Loss	Loss	No loss	No loss	MSI-H	+	+	+	+	+	MSI-H
67	+	+	+	+	+	MSI-H	+	—	+	+	+	MSI-H	0	0	95	100	Loss	Loss	No loss	No loss	MSI-H	Loss	Loss	No loss	No loss	MSI-H	Loss	Loss	No loss	No loss	MSI-H	+	—	+	+	+	MSI-H
68	+	+	+	—	+	MSI-H	+	+	+	+	+	MSI-H	0	0	100	90	Loss	Loss	No loss	No loss	MSI-H	Loss	Loss	No loss	No loss	MSI-H	Loss	Loss	No loss	No loss	MSI-H	+	+	+	+	+	MSI-H
69	+	+	+	+	+	MSI-H	+	+	+	+	+	MSI-H	0	0	80	100	Loss	Loss	No loss	No loss	MSI-H	Loss	Loss	No loss	No loss	MSI-H	Loss	Loss	No loss	No loss	MSI-H	+	+	+	+	+	MSI-H
70	+	+	+	—	+	MSI-H	+	+	+	+	+	MSI-H	100	100	0	0	No loss	No loss	Loss	Loss	MSI-H	No loss	No loss	Loss	Loss	MSI-H	No loss	No loss	Loss	Loss	MSI-H	+	+	+	+	+	MSI-H
71	+	+	+	+	+	MSI-H	+	+	+	+	+	MSI-H	0	0	100	100	Loss	Loss	No loss	No loss	MSI-H	Loss	Loss	No loss	No loss	MSI-H	Loss	Loss	No loss	No loss	MSI-H	+	—	+	—	+	MSI-H
72	+	+	+	+	+	MSI-H	+	+	+	+	+	MSI-H	100	95	3	3	No loss	No loss	No loss	No loss	MSS	No loss	No loss	No loss	No loss	MSS	No loss	No loss	Loss	Loss	MSI-H	+	+	+	+	+	MSI-H
73	+	+	+	+	+	MSI-H	+	+	+	+	+	MSI-H	0	0	100	100	Loss	Loss	No loss	No loss	MSI-H	Loss	Loss	No loss	No loss	MSI-H	Loss	Loss	No loss	No loss	MSI-H	+	+	+	+	+	MSI-H
74	+	+	+	+	+	MSI-H	+	—	+	+	+	MSI-H	0	0	90	80	Loss	Loss	No loss	No loss	MSI-H	Loss	Loss	No loss	No loss	MSI-H	Loss	Loss	No loss	No loss	MSI-H	+	+	+	+	+	MSI-H
75	+	+	+	+	+	MSI-H	+	+	+	+	+	MSI-H	4	4	100	95	No loss	No loss	No loss	No loss	MSS	No loss	No loss	No loss	No loss	MSS	Loss	Loss	No loss	No loss	MSI-H	+	+	+	+	+	MSI-H
76	+	+	+	+	+	MSI-H	+	+	+	+	+	MSI-H	0	0	80	95	Loss	Loss	No loss	No loss	MSI-H	Loss	Loss	No loss	No loss	MSI-H	Loss	Loss	No loss	No loss	MSI-H	+	+	+	+	+	MSI-H
77	—	—	—	—	—	MSS	—	—	—	—	—	MSS	100	100	100	100	No loss	No loss	No loss	No loss	MSS	No loss	No loss	No loss	No loss	MSS	No loss	No loss	No loss	No loss	MSS	—	—	—	—	—	MSS
78	—	—	—	—	—	MSS	—	—	—	—	—	MSS	100	100	100	100	No loss	No loss	No loss	No loss	MSS	No loss	No loss	No loss	No loss	MSS	No loss	No loss	No loss	No loss	MSS	—	—	—	—	—	MSS
79	—	—	—	—	—	MSS	—	—	—	—	—	MSS	100	100	100	100	No loss	No loss	No loss	No loss	MSS	No loss	No loss	No loss	No loss	MSS	No loss	No loss	No loss	No loss	MSS	—	—	—	—	—	MSS
80	—	—	—	—	—	MSS	—	—	—	—	—	MSS	100	100	100	100	No loss	No loss	No loss	No loss	MSS	No loss	No loss	No loss	No loss	MSS	No loss	No loss	No loss	No loss	MSS	—	—	—	—	—	MSS
81	—	—	—	—	—	MSS	—	—	—	—	—	MSS	100	100	100	100	No loss	No loss	No loss	No loss	MSS	No loss	No loss	No loss	No loss	MSS	No loss	No loss	No loss	No loss	MSS	—	—	—	—	—	MSS
82	—	—	—	—	—	MSS	—	—	—	—	—	MSS	100	100	100	100	No loss	No loss	No loss	No loss	MSS	No loss	No loss	No loss	No loss	MSS	No loss	No loss	No loss	No loss	MSS	—	—	—	—	—	MSS
83	—	—	—	—	—	MSS	—	—	—	—	—	MSS	100	100	100	100	No loss	No loss	No loss	No loss	MSS	No loss	No loss	No loss	No loss	MSS	No loss	No loss	No loss	No loss	MSS	—	—	—	—	—	MSS
84	—	—	—	—	—	MSS	—	—	—	—	—	MSS	100	100	100	100	No loss	No loss	No loss	No loss	MSS	No loss	No loss	No loss	No loss	MSS	No loss	No loss	No loss	No loss	MSS	—	—	—	—	—	MSS
85	—	—	—	—	—	MSS	—	—	—	—	—	MSS	100	100	100	100	No loss	No loss	No loss	No loss	MSS	No loss	No loss	No loss	No loss	MSS	No loss	No loss	No loss	No loss	MSS	—	—	—	—	—	MSS
86	—	—	—	—	—	MSS	—	—	—	+	—	MSI-L	100	100	100	100	No loss	No loss	No loss	No loss	MSS	No loss	No loss	No loss	No loss	MSS	No loss	No loss	No loss	No loss	MSS	—	—	—	—	—	MSS
87	—	—	—	—	—	MSS	—	—	—	—	—	MSS	100	100	100	100	No loss	No loss	No loss	No loss	MSS	No loss	No loss	No loss	No loss	MSS	No loss	No loss	No loss	No loss	MSS	—	—	—	—	—	MSS
88	—	—	—	—	—	MSS	—	—	—	—	—	MSS	100	100	100	100	No loss	No loss	No loss	No loss	MSS	No loss	No loss	No loss	No loss	MSS	No loss	No loss	No loss	No loss	MSS	—	—	—	—	—	MSS
89	—	—	—	—	—	MSS	—	—	—	—	—	MSS	100	100	100	100	No loss	No loss	No loss	No loss	MSS	No loss	No loss	No loss	No loss	MSS	No loss	No loss	No loss	No loss	MSS	—	—	—	—	—	MSS
90	—	—	—	—	—	MSS	—	—	—	—	—	MSS	100	100	100	100	No loss	No loss	No loss	No loss	MSS	No loss	No loss	No loss	No loss	MSS	No loss	No loss	No loss	No loss	MSS	—	—	—	—	—	MSS
91	—	—	—	—	—	MSS	—	—	—	—	—	MSS	100	100	100	100	No loss	No loss	No loss	No loss	MSS	No loss	No loss	No loss	No loss	MSS	No loss	No loss	No loss	No loss	MSS	—	—	—	—	—	MSS
92	—	—	—	—	—	MSS	—	—	—	—	—	MSS	100	100	100	100	No loss	No loss	No loss	No loss	MSS	No loss	No loss	No loss	No loss	MSS	No loss	No loss	No loss	No loss	MSS	—	—	—	—	—	MSS
93	—	—	—	—	—	MSS	—	—	—	—	—	MSS	100	100	100	100	No loss	No loss	No loss	No loss	MSS	No loss	No loss	No loss	No loss	MSS	No loss	No loss	No loss	No loss	MSS	—	—	—	—	—	MSS
94	—	—	—	—	—	MSS	—	—	—	—	—	MSS	100	100	100	100	No loss	No loss	No loss	No loss	MSS	No loss	No loss	No loss	No loss	MSS	No loss	No loss	No loss	No loss	MSS	—	—	—	—	—	MSS
95	—	—	—	—	—	MSS	—	—	—	—	—	MSS	100	100	100	100	No loss	No loss	No loss	No loss	MSS	No loss	No loss	No loss	No loss	MSS	No loss	No loss	No loss	No loss	MSS	—	—	—	—	—	MSS
96	—	—	—	—	—	MSS	—	—	—	—	—	MSS	100	100	100	100	No loss	No loss	No loss	No loss	MSS	No loss	No loss	No loss	No loss	MSS	No loss	No loss	No loss	No loss	MSS	—	—	—	—	—	MSS
97	—	—	—	—	—	MSS	—	—	—	—	—	MSS	100	100	100	100	No loss	No loss	No loss	No loss	MSS	No loss	No loss	No loss	No loss	MSS	No loss	No loss	No loss	No loss	MSS	—	—	—	—	—	MSS
98	—	—	—	—	—	MSS	—	—	—	—	—	MSS	100	100	100	100	No loss	No loss	No loss	No loss	MSS	No loss	No loss	No loss	No loss	MSS	No loss	No loss	No loss	No loss	MSS	—	—	—	—	—	MSS
99	—	—	—	—	—	MSS	—	—	—	—	—	MSS	100	100	100	100	No loss	No loss	No loss	No loss	MSS	No loss	No loss	No loss	No loss	MSS	No loss	No loss	No loss	No loss	MSS	+	—	—	—	—	MSI-L
100	—	—	—	—	—	MSS	—	—	—	—	—	MSS	100	100	100	100	No loss	No loss	No loss	No loss	MSS	No loss	No loss	No loss	No loss	MSS	No loss	No loss	No loss	No loss	MSS	—	—	—	—	—	MSS
101	—	—	—	—	—	MSS	—	—	—	—	—	MSS	100	100	100	100	No loss	No loss	No loss	No loss	MSS	No loss	No loss	No loss	No loss	MSS	No loss	No loss	No loss	No loss	MSS	—	—	—	—	—	MSS
102	—	—	—	—	—	MSS	—	—	—	—	—	MSS	100	100	100	100	No loss	No loss	No loss	No loss	MSS	No loss	No loss	No loss	No loss	MSS	No loss	No loss	No loss	No loss	MSS	+	—	—	—	—	MSI-L
103	—	—	—	—	—	MSS	—	—	—	—	—	MSS	100	100	100	100	No loss	No loss	No loss	No loss	MSS	No loss	No loss	No loss	No loss	MSS	No loss	No loss	No loss	No loss	MSS	—	—	—	—	—	MSS
104	—	—	—	—	—	MSS	+	—	—	—	—	MSI-L	100	100	100	100	No loss	No loss	No loss	No loss	MSS	No loss	No loss	No loss	No loss	MSS	No loss	No loss	No loss	No loss	MSS	—	—	—	—	—	MSS
105	—	—	—	—	—	MSS	—	—	—	—	—	MSS	100	100	100	100	No loss	No loss	No loss	No loss	MSS	No loss	No loss	No loss	No loss	MSS	No loss	No loss	No loss	No loss	MSS	—	—	—	—	—	MSS
106	—	—	—	—	—	MSS	—	—	—	—	—	MSS	100	100	100	100	No loss	No loss	No loss	No loss	MSS	No loss	No loss	No loss	No loss	MSS	No loss	No loss	No loss	No loss	MSS	—	—	—	—	—	MSS
107	—	—	—	—	—	MSS	—	—	—	—	—	MSS	100	100	100	100	No loss	No loss	No loss	No loss	MSS	No loss	No loss	No loss	No loss	MSS	No loss	No loss	No loss	No loss	MSS	—	—	—	—	—	MSS
108	—	—	—	—	—	MSS	—	—	—	—	—	MSS	100	100	100	100	No loss	No loss	No loss	No loss	MSS	No loss	No loss	No loss	No loss	MSS	No loss	No loss	No loss	No loss	MSS	—	—	—	—	—	MSS
109	—	—	—	—	—	MSS	—	—	—	—	—	MSS	100	100	100	100	No loss	No loss	No loss	No loss	MSS	No loss	No loss	No loss	No loss	MSS	No loss	No loss	No loss	No loss	MSS	—	—	—	—	—	MSS
110	—	—	—	—	—	MSS	—	—	—	—	—	MSS	100	100	100	100	No loss	No loss	No loss	No loss	MSS	No loss	No loss	No loss	No loss	MSS	No loss	No loss	No loss	No loss	MSS	—	—	—	—	—	MSS
111	—	—	—	—	—	MSS	—	—	—	—	—	MSS	100	100	100	100	No loss	No loss	No loss	No loss	MSS	No loss	No loss	No loss	No loss	MSS	No loss	No loss	No loss	No loss	MSS	—	—	—	—	—	MSS
112	—	—	—	—	—	MSS	—	—	—	—	—	MSS	100	100	100	100	No loss	No loss	No loss	No loss	MSS	No loss	No loss	No loss	No loss	MSS	No loss	No loss	No loss	No loss	MSS	—	—	—	—	—	MSS
113	—	—	—	—	—	MSS	—	—	—	—	—	MSS	100	100	100	100	No loss	No loss	No loss	No loss	MSS	No loss	No loss	No loss	No loss	MSS	No loss	No loss	No loss	No loss	MSS	—	—	—	—	—	MSS
114	—	—	—	—	—	MSS	—	—	—	—	—	MSS	100	100	100	100	No loss	No loss	No loss	No loss	MSS	No loss	No loss	No loss	No loss	MSS	No loss	No loss	No loss	No loss	MSS	—	—	—	—	—	MSS
115	—	—	—	—	—	MSS	—	—	—	—	—	MSS	100	100	100	100	No loss	No loss	No loss	No loss	MSS	No loss	No loss	No loss	No loss	MSS	No loss	No loss	No loss	No loss	MSS	—	—	—	—	—	MSS
116	—	—	—	—	—	MSS	—	—	—	—	—	MSS	100	100	100	100	No loss	No loss	No loss	No loss	MSS	No loss	No loss	No loss	No loss	MSS	No loss	No loss	No loss	No loss	MSS	—	—	—	—	—	MSS
117	—	—	—	—	—	MSS	—	—	—	—	—	MSS	100	100	100	100	No loss	No loss	No loss	No loss	MSS	No loss	No loss	No loss	No loss	MSS	No loss	No loss	No loss	No loss	MSS	—	—	—	—	—	MSS
118	—	—	—	—	—	MSS	—	—	—	—	—	MSS	100	100	100	100	No loss	No loss	No loss	No loss	MSS	No loss	No loss	No loss	No loss	MSS	No loss	No loss	No loss	No loss	MSS	—	—	—	—	—	MSS
119	—	—	—	—	—	MSS	—	—	—	—	—	MSS	100	100	100	100	No loss	No loss	No loss	No loss	MSS	No loss	No loss	No loss	No loss	MSS	No loss	No loss	No loss	No loss	MSS	—	—	—	+	—	MSI-L
120	—	—	—	—	—	MSS	—	—	—	—	—	MSS	100	100	100	100	No loss	No loss	No loss	No loss	MSS	No loss	No loss	No loss	No loss	MSS	No loss	No loss	No loss	No loss	MSS	—	—	—	—	—	MSS
121	—	—	—	—	—	MSS	—	—	—	—	—	MSS	100	100	100	100	No loss	No loss	No loss	No loss	MSS	No loss	No loss	No loss	No loss	MSS	No loss	No loss	No loss	No loss	MSS	—	—	—	—	—	MSS
122	—	—	—	—	—	MSS	—	—	—	—	—	MSS	100	100	100	100	No loss	No loss	No loss	No loss	MSS	No loss	No loss	No loss	No loss	MSS	No loss	No loss	No loss	No loss	MSS	—	—	—	—	—	MSS
123	—	—	—	—	—	MSS	—	—	—	—	—	MSS	100	100	100	100	No loss	No loss	No loss	No loss	MSS	No loss	No loss	No loss	No loss	MSS	No loss	No loss	No loss	No loss	MSS	—	—	—	—	—	MSS
124	—	—	—	—	—	MSS	—	—	—	—	—	MSS	100	100	100	100	No loss	No loss	No loss	No loss	MSS	No loss	No loss	No loss	No loss	MSS	No loss	No loss	No loss	No loss	MSS	—	—	—	—	—	MSS
125	—	—	—	—	—	MSS	—	—	—	—	—	MSS	100	100	100	100	No loss	No loss	No loss	No loss	MSS	No loss	No loss	No loss	No loss	MSS	No loss	No loss	No loss	No loss	MSS	—	—	—	—	—	MSS
126	—	—	—	—	—	MSS	—	—	—	—	—	MSS	100	100	100	100	No loss	No loss	No loss	No loss	MSS	No loss	No loss	No loss	No loss	MSS	No loss	No loss	No loss	No loss	MSS	—	—	—	—	—	MSS
127	—	—	—	—	—	MSS	—	—	—	—	—	MSS	100	100	100	100	No loss	No loss	No loss	No loss	MSS	No loss	No loss	No loss	No loss	MSS	No loss	No loss	No loss	No loss	MSS	—	—	—	—	—	MSS
128	—	—	—	—	—	MSS	—	—	—	—	—	MSS	100	100	100	100	No loss	No loss	No loss	No loss	MSS	No loss	No loss	No loss	No loss	MSS	No loss	No loss	No loss	No loss	MSS	—	—	—	+	—	MSI-L
129	—	—	—	—	—	MSS	—	—	—	—	—	MSS	100	100	100	100	No loss	No loss	No loss	No loss	MSS	No loss	No loss	No loss	No loss	MSS	No loss	No loss	No loss	No loss	MSS	—	—	—	—	—	MSS
130	—	—	—	—	—	MSS	—	—	—	—	—	MSS	100	100	100	100	No loss	No loss	No loss	No loss	MSS	No loss	No loss	No loss	No loss	MSS	No loss	No loss	No loss	No loss	MSS	—	—	—	—	—	MSS
131	—	—	—	—	—	MSS	—	—	—	—	—	MSS	100	100	100	100	No loss	No loss	No loss	No loss	MSS	No loss	No loss	No loss	No loss	MSS	No loss	No loss	No loss	No loss	MSS	—	—	—	—	—	MSS
132	—	—	—	—	—	MSS	—	—	—	—	—	MSS	100	100	100	100	No loss	No loss	No loss	No loss	MSS	No loss	No loss	No loss	No loss	MSS	No loss	No loss	No loss	No loss	MSS	—	—	—	—	—	MSS
133	—	—	—	—	—	MSS	—	—	—	—	—	MSS	100	100	100	100	No loss	No loss	No loss	No loss	MSS	No loss	No loss	No loss	No loss	MSS	No loss	No loss	No loss	No loss	MSS	—	—	—	—	—	MSS
134	—	—	—	—	—	MSS	—	—	—	—	—	MSS	100	100	100	100	No loss	No loss	No loss	No loss	MSS	No loss	No loss	No loss	No loss	MSS	No loss	No loss	No loss	No loss	MSS	—	—	—	—	—	MSS
135	—	—	—	—	—	MSS	—	—	—	—	—	MSS	100	100	100	100	No loss	No loss	No loss	No loss	MSS	No loss	No loss	No loss	No loss	MSS	No loss	No loss	No loss	No loss	MSS	—	—	—	—	—	MSS
136	—	—	—	—	—	MSS	—	—	—	—	—	MSS	100	100	100	100	No loss	No loss	No loss	No loss	MSS	No loss	No loss	No loss	No loss	MSS	No loss	No loss	No loss	No loss	MSS	—	—	—	—	—	MSS
137	—	—	—	—	—	MSS	—	—	—	—	—	MSS	100	100	100	100	No loss	No loss	No loss	No loss	MSS	No loss	No loss	No loss	No loss	MSS	No loss	No loss	No loss	No loss	MSS	—	—	—	—	—	MSS
138	—	—	—	—	—	MSS	—	—	—	—	—	MSS	100	100	100	100	No loss	No loss	No loss	No loss	MSS	No loss	No loss	No loss	No loss	MSS	No loss	No loss	No loss	No loss	MSS	—	—	—	—	—	MSS
139	—	—	—	—	—	MSS	—	—	—	—	—	MSS	100	100	100	100	No loss	No loss	No loss	No loss	MSS	No loss	No loss	No loss	No loss	MSS	No loss	No loss	No loss	No loss	MSS	—	—	—	—	—	MSS
140	—	—	—	—	—	MSS	—	—	—	—	—	MSS	100	100	100	100	No loss	No loss	No loss	No loss	MSS	No loss	No loss	No loss	No loss	MSS	No loss	No loss	No loss	No loss	MSS	—	—	—	—	—	MSS
141	—	—	—	—	—	MSS	—	—	—	—	—	MSS	100	100	100	100	No loss	No loss	No loss	No loss	MSS	No loss	No loss	No loss	No loss	MSS	No loss	No loss	No loss	No loss	MSS	—	—	—	—	—	MSS
142	—	—	—	—	—	MSS	—	—	—	—	—	MSS	100	100	100	100	No loss	No loss	No loss	No loss	MSS	No loss	No loss	No loss	No loss	MSS	No loss	No loss	No loss	No loss	MSS	—	—	—	—	—	MSS
143	—	—	—	—	—	MSS	—	—	—	—	—	MSS	100	100	100	100	No loss	No loss	No loss	No loss	MSS	No loss	No loss	No loss	No loss	MSS	No loss	No loss	No loss	No loss	MSS	—	—	—	—	—	MSS
144	—	—	—	—	—	MSS	—	—	—	—	—	MSS	100	100	100	100	No loss	No loss	No loss	No loss	MSS	No loss	No loss	No loss	No loss	MSS	No loss	No loss	No loss	No loss	MSS	—	—	—	—	—	MSS
145	—	—	—	—	—	MSS	—	—	—	—	—	MSS	100	100	100	100	No loss	No loss	No loss	No loss	MSS	No loss	No loss	No loss	No loss	MSS	No loss	No loss	No loss	No loss	MSS	—	—	—	—	—	MSS
146	—	—	—	—	—	MSS	—	—	—	—	—	MSS	100	100	100	100	No loss	No loss	No loss	No loss	MSS	No loss	No loss	No loss	No loss	MSS	No loss	No loss	No loss	No loss	MSS	—	—	—	—	—	MSS
147	—	—	—	—	—	MSS	—	—	—	—	—	MSS	100	100	100	100	No loss	No loss	No loss	No loss	MSS	No loss	No loss	No loss	No loss	MSS	No loss	No loss	No loss	No loss	MSS	—	—	—	—	—	MSS
148	—	—	—	—	—	MSS	—	—	—	—	—	MSS	100	100	100	100	No loss	No loss	No loss	No loss	MSS	No loss	No loss	No loss	No loss	MSS	No loss	No loss	No loss	No loss	MSS	—	—	—	—	—	MSS
149	—	—	—	—	—	MSS	—	—	—	—	—	MSS	100	100	100	100	No loss	No loss	No loss	No loss	MSS	No loss	No loss	No loss	No loss	MSS	No loss	No loss	No loss	No loss	MSS	—	—	—	—	—	MSS
150	—	—	—	—	—	MSS	—	—	—	—	—	MSS	100	100	100	100	No loss	No loss	No loss	No loss	MSS	No loss	No loss	No loss	No loss	MSS	No loss	No loss	No loss	No loss	MSS	—	—	—	—	—	MSS
151	—	—	—	—	—	MSS	—	—	—	—	—	MSS	100	100	100	100	No loss	No loss	No loss	No loss	MSS	No loss	No loss	No loss	No loss	MSS	No loss	No loss	No loss	No loss	MSS	—	—	—	+	—	MSI-L
152	—	—	—	—	—	MSS	—	—	—	—	—	MSS	100	100	100	100	No loss	No loss	No loss	No loss	MSS	No loss	No loss	No loss	No loss	MSS	No loss	No loss	No loss	No loss	MSS	—	—	—	—	—	MSS
153	—	—	—	—	—	MSS	—	—	—	—	—	MSS	100	100	100	100	No loss	No loss	No loss	No loss	MSS	No loss	No loss	No loss	No loss	MSS	No loss	No loss	No loss	No loss	MSS	—	—	—	—	—	MSS
154	—	—	—	—	—	MSS	—	—	—	—	—	MSS	100	100	100	100	No loss	No loss	No loss	No loss	MSS	No loss	No loss	No loss	No loss	MSS	No loss	No loss	No loss	No loss	MSS	—	—	—	—	—	MSS
155	—	—	—	—	—	MSS	—	—	—	—	—	MSS	100	100	100	100	No loss	No loss	No loss	No loss	MSS	No loss	No loss	No loss	No loss	MSS	No loss	No loss	No loss	No loss	MSS	—	—	—	—	—	MSS
156	—	—	—	—	—	MSS	—	—	—	—	—	MSS	100	100	100	100	No loss	No loss	No loss	No loss	MSS	No loss	No loss	No loss	No loss	MSS	No loss	No loss	No loss	No loss	MSS	—	—	—	—	—	MSS
157	—	—	—	—	—	MSS	—	—	—	—	—	MSS	100	100	100	100	No loss	No loss	No loss	No loss	MSS	No loss	No loss	No loss	No loss	MSS	No loss	No loss	No loss	No loss	MSS	—	—	—	—	—	MSS
158	—	—	—	—	—	MSS	—	—	—	—	—	MSS	100	100	100	100	No loss	No loss	No loss	No loss	MSS	No loss	No loss	No loss	No loss	MSS	No loss	No loss	No loss	No loss	MSS	—	—	—	—	—	MSS
159	—	—	—	—	—	MSS	—	—	—	—	—	MSS	100	100	100	100	No loss	No loss	No loss	No loss	MSS	No loss	No loss	No loss	No loss	MSS	No loss	No loss	No loss	No loss	MSS	—	—	—	—	—	MSS
160	—	—	—	—	—	MSS	—	—	—	—	—	MSS	100	100	100	100	No loss	No loss	No loss	No loss	MSS	No loss	No loss	No loss	No loss	MSS	No loss	No loss	No loss	No loss	MSS	—	—	—	—	—	MSS
161	—	—	—	—	—	MSS	—	—	—	—	—	MSS	100	100	100	100	No loss	No loss	No loss	No loss	MSS	No loss	No loss	No loss	No loss	MSS	No loss	No loss	No loss	No loss	MSS	—	—	—	—	—	MSS
162	—	—	—	—	—	MSS	—	—	—	—	—	MSS	100	100	100	100	No loss	No loss	No loss	No loss	MSS	No loss	No loss	No loss	No loss	MSS	No loss	No loss	No loss	No loss	MSS	—	—	—	—	—	MSS
163	—	—	—	—	—	MSS	—	—	—	—	—	MSS	100	100	100	100	No loss	No loss	No loss	No loss	MSS	No loss	No loss	No loss	No loss	MSS	No loss	No loss	No loss	No loss	MSS	—	—	—	—	—	MSS
164	—	—	—	—	—	MSS	—	—	—	—	—	MSS	100	100	100	100	No loss	No loss	No loss	No loss	MSS	No loss	No loss	No loss	No loss	MSS	No loss	No loss	No loss	No loss	MSS	—	—	—	—	—	MSS
165	—	—	—	—	—	MSS	—	—	—	—	—	MSS	100	100	100	100	No loss	No loss	No loss	No loss	MSS	No loss	No loss	No loss	No loss	MSS	No loss	No loss	No loss	No loss	MSS	—	—	—	—	—	MSS
166	—	—	—	—	—	MSS	—	—	—	—	—	MSS	100	100	100	100	No loss	No loss	No loss	No loss	MSS	No loss	No loss	No loss	No loss	MSS	No loss	No loss	No loss	No loss	MSS	—	—	—	—	—	MSS
