# Supplementary material for: Microsatellite instability test using peptide nucleic acid probe-mediated melting point analysis: a comparison study
Source: BMC Cancer. 2018 Dec 4;18:1218. doi: 10.1186/s12885-018-5127-6 (PMC6280403; doi:10.1186/s12885-018-5127-6)
Supplement: Supplementary file 3 — Table S2. Sensitivity of MNR and PNA method. Table S3. MSI-H detection rate for each marker used in PNA and MNR method. Table S4. Clinicopathologic characteristics of 76 MSI-H CRC patients and 90 MSS CRC patients. Table S5. Clinicopathologic characteristics of patients with MSI-H CRCs associated with sporadic conditions or Lynch syndrome. Table S6. IHC-mediated MSI-H detection rate depending on the combination of markers and variable cut-off values for loss of MMR protein expression. (PDF 342 kb) [file 12885_2018_5127_MOESM3_ESM.pdf]

Supplementary Table S2 Sensitivity of MNR and PNA method

Variant (%)	MNR method					Result
	NR21	***NR24***	***NR27***	***BAT25***	***BAT26***	**MSS/MSI-H**
5%	–	–	–	–	–	MSS
10%	O	–	O	–	–	MSI-H
20%	O	O	O	O	O	MSI-H
40%	O	O	O	O	O	MSI-H
100%	O	O	O	O	O	MSI-H
**Variant (%)**	**PNA method**					**Result**
	NR21	***NR24***	***NR27***	***BAT25***	***BAT26***	**MSS/MSI-H**
1%	O	–	–	O	–	MSI-H
5%	O	O	O	O	O	MSI-H
10%	O	O	O	O	O	MSI-H
20%	O	O	O	O	O	MSI-H
100%	O	O	O	O	O	MSI-H

Supplementary Table S3 MSI-H detection rate for each marker used in PNA and MNR method

Marker	PNA method (n = 76)		MNR method (n = 76)		p-value*
	Number of samples	Detection rate (%)	Number of samples	Detection rate (%)	
NR21	74	97.37%	71	93.42%	0.375
NR24	71	93.42%	64	84.21%	0.039
BAT26	71	93.42%	63	82.89%	0.077
BAT25	72	94.74%	72	94.74%	1
NR27	75	98.78%	74	97.37%	1

* McNemar analysis was used to compare two different test results in the same cases.

Supplementary Table S4 Clinicopathologic characteristics of 76 MSI-H CRC patients and 90 MSS CRC patients

Clinicopathologic factor	Classification	MSI-H (n=76)		MSS (n=90)		*p*-value*
Age, years		54.39±15.11		61.16±12.61		0.002
Gender	Female	26	34.2%	34	37.8%	0.634
	Male	50	65.8%	56	62.2%	
Location	Right side	53	69.7%	25	27.8%	<0.01
	Left side	23	30.3%	65	72.2%	
Pre OP CEA	≤5ng/ml	68	89.5%	61	67.8%	0.001
	>5ng/ml	8	10.5%	29	32.2%	
Gross type	Non-exophytic	18	23.7%	35	38.9%	0.036
	Exophytic	58	76.3%	55	61.1%	
Size (cm)		6.13±2.8		4.43±2.05		<0.001
Histologic diagnosis	Adenocarcinoma	63	82.9%	84	93.3%	0.031
	Mucinous adenocarcinoma	11	14.5%	5	5.6%	
	Signet ring cell carcinoma	2	2.6%	0	0.0%	
	Neuroendocrine carcinoma	0	0.0%	1	1.2%	
T stage	pT1	2	2.6%	10	11.1%	0.002
	pT2	13	17.1%	13	14.4%	
	pT3	57	75.0%	48	53.3%	
	pT4a and pT4b	4	5.3%	19	21.1%	

* Fisher's exact test and Mann-Whitney test were used for the statistical analysis of categorical variables and continuous variables, respectively.

Supplementary Table S5 Clinicopathologic characteristics of patients with MSI-H CRCs associated with sporadic conditions or Lynch syndrome

Clinicopathologic factor	Classification	MSI-H CRCs associated with sporadic condition (n=67)		MSI-H CRCs associated with Lynch syndrome (n=9)		*p*-value*
Age, years		56.50±14.37		38.66±11.10		0.001
Gender	Female	24	35.8%	2	22.2%	0.488
	Male	43	64.2%	7	77.8%	
Location	Right side	49	73.1%	4	44.4%	0.119
	Left side	18	26.9%	5	55.6%	
Pre OP CEA	≤5ng/ml	59	88.1%	8	88.9%	1
	>5ng/ml	8	11.9%	1	11.1%	
Gross type	Non-exophytic	16	23.9%	2	22.2%	1
	Exophytic	51	76.1%	7	77.8%	
Size		6.36±2.80		4.34±2.12		0.041
Histologic diagnosis	Adenocarcinoma	56	83.6%	7	77.8%	0.321
	Mucinous carcinoma	10	14.9%	1	11.1%	
	Signet ring cell carcinoma	1	1.5%	1	11.1%	
T stage	pT1	2	3.0%	0	0.0%	0.644
	pT2	11	16.4%	2	22.2%	
	pT3	50	74.6%	7	77.8%	
	pT4a and pT4b	4	6.0%	0	0.0%	
Lymphovascular invasion	Absent	50	75.8%	6	66.7%	0.684
	Present	16	24.2%	3	33.3%	
Mucin formation	Absent	27	40.9%	5	42.7%	0.789
	Focal	30	45.5%	3	44.0%	
	Predominant	9	13.6%	1	13.3%	
Invasion pattern	Expanding	36	55.4%	4	44.4%	0.724
	Infiltrative	29	44.6%	5	55.6%	
Crohn-like reaction	Absent	25	38.5%	2	22.2%	0.472
	Present	40	61.5%	7	77.8%	
Tumor budding	Absent	63	96.9%	8	88.9%	0.326
	Present	2	3.1%	1	11.1%	
MMR protein expression by IHC	MLH1/PMS2 loss	42	62.7%	7	77.8%	1
	MSH2/MSH6 loss	15	22.4%	2	22.2%	
	PMS2 loss	1	1.5%	0	0.0%	
	MSH6 loss	5	7.5%	0	0.0%	
	MLH1/PMS2/MSH2/MSH6 loss	1	1.5%	0	0.0%	
	MLH1/MSH2/MSH6 loss	1	1.5%	0	0.0%	

	MLH1/PMS2/MSH6 loss	1	1.5%	0	0.0%	
	No loss	1	1.5%	0	0.0%	
Instablilty of MSI makers in PNA-PCR	NR21	65	97.00%	8	88.90%	0.319
	NR24	63	94.00%	9	100%	1
	BAT26	62	92.50%	9	100%	1
	BAT25	64	95.50%	9	100%	1
	NR27	66	98.50%	9	100%	1
Instablilty of MSI makers in PCR-MNR	NR21	63	94.00%	9	100%	1
	NR24	55	82.10%	8	88.90%	1
	BAT26	54	80.60%	9	100%	0.344
	BAT25	63	94.00%	8	88.90%	0.477
	NR27	65	97.00%	9	100%	1

* Fisher's exact test and Mann-Whitney test were used for the statistical analysis of categorical variables and continuous variables, respectively.

Supplementary Table S6 IHC-mediated MSI-H detection rate depending on the combination of markers and variable cut-off values for loss of MMR protein expression

Combination of MMR markers	MSI-H detection rate (n = 76)					
	Cut off value: complete loss		Cut off value: ≥99% loss		Cut off value: ≥95% loss	
	Number of samples	Detection rate (%)	Number of samples	Detection rate (%)	Number of samples	Detection rate (%)
**MLH1**	50	65.79%	50	65.79%	51	67.11%
**MSH2**	15	19.74%	15	19.74%	19	25.00%
**PMS2**	48	63.16%	51	67.11%	52	68.42%
**MSH6**	8	10.53%	17	22.37%	25	32.89%
**MLH1 and MSH2**	64	84.21%	64	84.21%	69	90.79%
**PMS2 and MSH6**	56	73.68%	68	89.47%	75	98.68%
**MLH1, PMS2, MSH2, and MSH6**	69	90.79%	73	96.05%	75	98.68%
